# Mambalgin-2 Inhibits Lung Adenocarcinoma Growth and Migration by Selective Interaction With ASIC1/α-ENaC/γ-ENaC Heterotrimer

**DOI:** 10.3389/fonc.2022.904742

**Published:** 2022-06-28

**Authors:** Anastasia V. Sudarikova, Maxim L. Bychkov, Dmitrii S. Kulbatskii, Vladislav I. Chubinskiy-Nadezhdin, Olga V. Shlepova, Mikhail A. Shulepko, Sergey G. Koshelev, Mikhail P. Kirpichnikov, Ekaterina N. Lyukmanova

**Affiliations:** ^1^ Laboratory of Bioengineering of Neuromodulators and Neuroreceptors, Shemyakin-Ovchinnikov Institute of Bioorganic Chemistry, Russian Academy of Sciences, Moscow, Russia; ^2^ Group of Ionic Mechanisms of Cell Signaling, Department of Intracellular Signaling and Transport, Institute of Cytology, Russian Academy of Sciences, St. Petersburg, Russia; ^3^ Phystech School of Biological and Medical Physics, Moscow Institute of Physics and Technology (National Research University), Dolgoprudny, Russia; ^4^ Laboratory of Neuroreceptors and Neuroregulators, Shemyakin-Ovchinnikov Institute of Bioorganic Chemistry, Russian Academy of Sciences, Moscow, Russia; ^5^ Interdisciplinary Scientific and Educational School of Moscow University «Molecular Technologies of the Living Systems and Synthetic Biology», Faculty of Biology, Lomonosov Moscow State University, Moscow, Russia

**Keywords:** acid-sensing ion channel, degenerin/epithelial Na^+^ channel, lung adenocarcinoma, mambalgin-2, lung cancer therapy

## Abstract

Lung cancer is one of the most common cancer types in the world. Despite existing treatment strategies, overall patient survival remains low and new targeted therapies are required. Acidification of the tumor microenvironment drives the growth and metastasis of many cancers. Acid sensors such as acid-sensing ion channels (ASICs) may become promising targets for lung cancer therapy. Previously, we showed that inhibition of the ASIC1 channels by a recombinant analogue of mambalgin-2 from *Dendroaspis polylepis* controls oncogenic processes in leukemia, glioma, and melanoma cells. Here, we studied the effects and molecular targets of mambalgin-2 in lung adenocarcinoma A549 and Lewis cells, lung transformed WI-38 fibroblasts, and lung normal HLF fibroblasts. We found that mambalgin-2 inhibits the growth and migration of A549, metastatic Lewis P29 cells, and WI-38 cells, but not of normal fibroblasts. A549, Lewis, and WI-38 cells expressed different ASIC and ENaC subunits, while normal fibroblasts did not at all. Mambalgin-2 induced G2/M cell cycle arrest and apoptosis in lung adenocarcinoma cells. In line, acidification-evoked inward currents were observed only in A549 and WI-38 cells. Gene knockdown showed that the anti-proliferative and anti-migratory activity of mambalgin-2 is dependent on the expression of ASIC1a, α-ENaC, and γ-ENaC. Using affinity extraction and immunoprecipitation, mambalgin-2 targeting of ASIC1a/α-ENaC/γ-ENaC heteromeric channels in A549 cells was shown. Electrophysiology studies in *Xenopus* oocytes revealed that mambalgin-2 inhibits the ASIC1a/α-ENaC/γ-ENaC channels with higher efficacy than the ASIC1a channels, pointing on the heteromeric channels as a primary target of the toxin in cancer cells. Finally, bioinformatics analysis showed that the increased expression of ASIC1 and γ-ENaC correlates with a worse survival prognosis for patients with lung adenocarcinoma. Thus, the ASIC1a/α-ENaC/γ-ENaC heterotrimer can be considered a marker of cell oncogenicity and its targeting is promising for the design of new selective cancer therapeutics.

## Introduction

Lung cancer is one of the most common cancer types in the world with the highest frequency of disease‐associated morbidity and mortality. According to the cancer statistics from the Global Cancer Observatory (GLOBOCAN) in 2020, lung cancer accounts for 2.2 million newly diagnosed cases and 1.8 million deaths (18% of total cancer deaths) (1). Approximately 85% of all lung cancer diagnostic cases are non–small cell lung cancer (NSCLC), such as lung adenocarcinoma (2). Despite the existing treatment strategies for this disease such as chemotherapy, radiotherapy, and surgery, the overall patient survival rate remains low. Therefore, it is important to develop novel therapies and to search for specific targets to substantially improve the treatment and survival prognosis for patients with lung adenocarcinoma.

Tumor progression is accompanied by inflammatory processes, immunosuppression, hypoxia, and acidification of the microenvironment (3, 4). Acidosis of the microenvironment induced by local hypoxia and enhanced glycolytic activity is one of the biochemical characteristics of tumors. The local extracellular pH value may drop to 6.2–6.9 in solid tumors (4, 5) and to pH 5.5 or lower in chronic inflammatory conditions (6). Low extracellular pH was shown to promote progression, invasion, and metastasis of the tumors (5, 7, 8).

Different types of ion channels are involved in the regulation of many physiological and pathological functions in cells and tissues. Among them, acid-sensing ion channels (ASICs) contribute to cellular response to the altered pH in the tumor environment due to their ability to be activated by low extracellular pH. ASICs are proton-gated cation channels and belong to the degenerin/epithelial Na^+^ channel (DEG/ENaC) superfamily (9). Currently, at least four genes encoding six types of the ASIC subunits are known: ASIC1a, ASIC1b, ASIC2a, ASIC2b, ASIC3, ASIC4 (10). ENaCs are sodium-selective channels that are expressed in a variety of epithelial and non-epithelial cells and tissues (11). Currently, the four subunits (α-ENaC, β-ENaC, γ-ENaC, and δ-ENaC) are identified. Usually they form the heteromeric channels (12, 13). ASIC subunits alone or in combination with ENaC subunits can form functionally active homo- or heterotrimeric channels with different physiological and biophysical properties. For example, the hybrid channels formed by ASIC1 and ENaCs were reported (14). In addition to a high level of ASIC expression in the nervous system, where they are involved in physiological processes such as pain perception, synaptic plasticity, learning, and memory, most ASIC and ENaC subunits are present in different non-excitable tissues, and the channels formed by them can also be implicated in tumor pathogenesis. Our previous results and literature data point on the possible ASIC1 and ENaC expressions in leukemia (15), glioma (16), melanoma (17), breast cancer (18), and pancreatic (19), lung (3), and hepatocellular carcinoma cells (20), where the ASIC1-containing channels participate in the regulation of cancer cell proliferation, migration, and invasion. In particular, the ASIC1a-containing channels affect the proliferation and migration of lung adenocarcinoma A549 cells induced by extracellular acidosis (3). However, the detailed molecular mechanisms of the ASIC1 oncogenic effects in lung adenocarcinoma as well as the prospects of the selective ASIC1 targeting for lung cancer therapy remain unclear.

Amiloride and psalmotoxin from the venom of the tarantula *Psalmopoeus cambridgei* (PcTx1) are the best known inhibitors of ASIC1a (21), and their antiproliferative effects on several types of tumor cells, including glioma (22) and carcinoma (3, 18), were reported. PcTx1 is the only specific inhibitor of the homomeric ASIC1a channel, but it is not suitable for clinical use, since it can inhibit and potentiate the heteromeric ASIC1a/ASIC2a channels upon different conditions, and its action is dependent on the pH value (23). Amiloride, which is widely used to inhibit the ASIC1 channels, demonstrates low selectivity and is more specific for the ENaC channels (24). Mambalgins isolated from the venom of black mamba *Dendroaspis polylepis* are three-finger small proteins from the Ly6/uPAR family (25), which reversibly selectively inhibit both the homomeric (ASIC1a) and heteromeric (ASIC1a /2a, ASIC1a /2b) channels containing the ASIC1a subunit (26). Previously, we demonstrated that the selective blocking of the amiloride-sensitive ASIC1a-containing channels by recombinant mambalgin-2 inhibits the proliferation of leukemia (15) and glioblastoma cells (16) causing the cell cycle arrest in the G1 phase and apoptosis, without affecting the proliferation of normal astrocytes.

Here, we studied mambalgin-2’s effects on the growth and migration of human lung adenocarcinoma A549 cells, murine parental and metastatic lung adenocarcinoma Lewis cells, human lung immortalized fibroblasts WI-38, and human lung primary normal fibroblasts HLF-140. Also, we analyzed the expression profile of various ASIC and ENaC subunits and activation of the acid-sensitive channels in the cells in the absence and presence of mambalgin-2. Results obtained together with the affinity purification data and electrophysiology study in *X. laevis* oocytes allowed us to identify and characterize the new selective target of mambalgin-2 in cancer cells—the heteromeric ASIC1a/α-ENaC/γ-ENaC channels. Bioinformatic analysis revealed a correlation between the increased expression of mRNAs coding the ASIC1 and γ-ENaC subunits with the worse survival prognosis for the patients with lung adenocarcinoma. Data obtained point on the heteromeric ASIC1a/α-ENaC/γ-ENaC channels as a promising target for new lung cancer therapies.

## Materials and Methods

### Mambalgin-2 Production and Characterization

Recombinant mambalgin-2 was produced in *E. coli* as described previously (27). The purity and homogeneity of the recombinant protein (> 95%) were confirmed by HPLC, MALDI-MS, and SDS-PAGE. Disulfide bond formation was confirmed in the reaction with Ellman’s reagent (Sigma-Aldrich, Saint-Louis, MO, USA). The correct spatial structure of the recombinant protein was confirmed by 1D ^1^H-NMR-spectroscopy.

### Cell Cultures

Human lung carcinoma A549 cell line and pseudo-normal human embryonic lung fibroblasts WI-38 VA 13 subline 2RA (transformed by SV-40 to increase the number of passages) were obtained from the shared research facility “Vertebrate cell culture collection” (Institute of Cytology, St. Petersburg, Russia). A549 cells were cultured in DMEM medium from Biolot (St. Petersburg, Russia), 10% FBS (Biowest, Nuaillé, France), and 80 mg/mL gentamicin, and WI-38 in EMEM medium (Biolot, St. Petersburg, Russia) supplemented with 1% non-essential amino acids (NEAA, Thermo Fisher Sci, Waltham, MA, USA), 10% FBS, and 80 mg/mL gentamycin. Primary normal embryo human lung fibroblasts HLF-104 (designated below as HLF) cells (Biolot) were passaged in EMEM medium (Biolot) containing 10% FBS and 80 mg/mL gentamycin. Murine Lewis lung adenocarcinoma cells and its metastatic subline P29 were a kind gift from Dr. B.S. Akopov and were cultivated in DMEM supplemented with 10% FBS. Cells were maintained at 37°C in a humidified atmosphere with 5% CO_2_. All types of the cells were subcultured twice per week. Cells were passaged no more than 40 times and regularly tested for the absence of mycoplasma contamination by the PCR kit (MycoReport, Evrogen, Moscow, Russia).

### Cell and Spheroid Proliferation Assay

To assay the influence of the medium acidification on the cell growth, the cells were seeded in 96-well culture plates (5 × 10^3^ cells per well) and grown for 24 h. After that, the medium was aspirated and changed to the fresh one with pH 7.4 (normal medium) or 5.5 (containing 10 mM MES, acidic medium) and cells were incubated for 72 h without the medium change. To study mambalgin-2’s influence on the cell proliferation, the cells were seeded in 96-well cell culture plates (5 × 10^3^ cells/per well) in the normal or acidic medium and grown for 24 h. Thereafter, mambalgin-2 (from the 10 mM stock solution in 100% DMSO) was dissolved in the normal or acidic medium and added to the cells at concentrations from 10^-11^ to 10^-5^ M for further incubation during 72 h without the medium change. The maximal DMSO concentration did not exceed 0.1%. The added DMSO did not influence the cell growth as was established in additional experiments. For spheroid reconstruction, A549 cells (1 × 10^3^ cells per well) were seeded on 96-well plates treated by poly(2-hydroxyethyl methacrylate) (Sigma-Aldrich) at pH 5.5 and incubated with 1 µM of mambalgin-2 for 24 h.

To analyze the cell viability of cells and spheroids, we used the WST-1 colorimetric test as described earlier (28). Briefly, WST-1 (water-soluble tetrazolium salt 1; Santa Cruz, Dallas, TX, USA) and 1-m-PMS (1-methoxy-5-methylphenazinium methyl sulfate, Santa Cruz) were added to the cells in concentrations of 0.25 mM and 5 μM, respectively, for 1 h, and formation of the colored product was measured at 450 nm with background subtraction at 655 nm on microplate reader Bio-Rad 680 (Bio-Rad, Hercules, CA, USA). The data were normalized to averaged readout from the control wells containing the cells without added compounds. The concentration–effect curves were fitted in the GraphPad Prism 8.0 software (GraphPad Software, San Diego, CA, USA). For investigation of the influence of medium acidification on the cells’ viability, the cells cultivated at pH 7.4 (normal medium) were used as control. For visualization of spheroids, they were aspirated from wells, stained with 0.4% Trypan Blue (PanEco, Moscow, Russia), and imaged using the CellDrop Brightfield Cell Counter (DeNovix, Wilmington, DE, USA).

### Gene Silencing

To block the expression of native ASIC1a, α-ENaC, and γ-ENaC, A549 cells were transfected with corresponding siRNA (**Table S1**, Synthol, Russia). Cells were seeded in six-well culture plates (2 × 10^5^ cells/well) and grown for 24 h. Then, three siRNAs to different parts of the corresponding genes were mixed (1 μg per well), and the mixture was diluted in 100 μl of the transfection buffer (Pan-Biotech, Aidenbach, Germany), incubated for 5 min, and mixed with 15 μl of the pre-diluted PANFect A-plus transfection reagent (Pan-Biotech). The final mixture was incubated for 30 min and added to A549 cells. The cells were incubated in a CO_2_ incubator for 4 h, and the cell media were replaced by the fresh one. After 96 h of incubation, the cells were detached by Versene and divided into two parts. The first part was incubated with ASIC1a (sheep, 1:1,000, ABIN350049, Antibodies-Online, Aachen, Germany), α-ENaC (rabbit, 1:1,000, ABIN1841945, Antibodies-Online), or γ-ENaC (mouse, 1:1,000, ABIN1865926, Antibodies-Online), washed, and incubated with the secondary TRITC-conjugated antibodies (713-025-003, 111-025-003, and 615-025-214, Jackson ImmunoResearch, West Grove, USA, for ASIC1a, α-ENaC, and γ-ENaC, respectively). The expression of the surface receptors was analyzed using the Attune NxT Flow Cytometer (Life Technologies, Carlsbad, CA, USA) for confirmation of gene knockdown (**Figure S1**). The second part of the cells was seeded in 96-well culture plates (5 × 10^4^ cells per well) at pH 7.4 or 5.5 and incubated with different concentrations of mambalgin-2 for 72 h without media change, and the WST-1 assay was performed as described above.

### “Wound-Healing” Migration Assay

To form the experimental wounds, cells were seeded into Ibidi Culture-Inserts 2 Well (Ibidi, Gräfelfing, Germany, 4–5 × 10^4^ cells/well) installed in 24-well culture plates. The cells were placed in a cell culture incubator with 37°C and 5% CO_2_ overnight to allow spreading. Before the experiments, the inserts were removed, and the culture medium was replaced with the normal or acidic fresh medium with or without 1 μM of mambalgin-2 (from the 10 mM stock solution in 100% DMSO) or equal DMSO amount. The phase-contrast images of the initial gaps were captured under an inverted microscope (10× objective, MicroMed, St. Petersburg, Russia) using the Toupcam 5.1 MPix CMOS Camera (ToupTek Photonics, Hangzhou, China) controlled by the ToupView 3.7 software (ToupTek Photonics). Then, the images of the same gaps were captured after 24 h from the start of the experiment. The gap sizes were calculated manually using the “Measure Area” tool in the ImageJ software (NIH, Bethesda, MD, USA) and normalized to the size of the initial gap (0 h). The area occupied by the migrated cells (in % from the initial gaps) was compared between groups.

### Real-Time PCR

Total RNA was isolated using the Bio-Rad Aurum RNA isolation kit (Bio-Rad) according to the manufacturer’s instructions. cDNA was synthesized by the Mint reverse transcriptase kit (Evrogen). After that, qPCR was performed with the ready-to-use SYBR Green HS mix (Evrogen) and primers specific to the *ACCN2* (coding the ASIC1a isoform, PubMed Gene ID NM_020039.4), *ACCN1*, *ACCN3*, *ACCN4*, *SCNN1A*, and *SCNN1G* genes (**Table S2**) using the Roche LightCycler 96 amplifier (Roche, Basel Switzerland). Data were analyzed by the ΔΔCt method and LightCycler SW software (Roche), and the gene expression was normalized to the expression of *β-ACTIN*, *GPDH*, and *RPL13a* housekeeping genes.

### Cell Cycle Analysis

For analysis of mambalgin-2’s influence on the cell cycle, the A549 and metastatic Lewis cells were seeded in six-well culture plates (2 × 10^5^ cells per well) at pH 5.5, grown for 24 h, and incubated with 1 μM mambalgin-2 for 72 h without the media change. Then the cells were detached from the wells by 0.5% trypsin-EDTA, washed with Earle’s Balanced Salt Solution (EBSS), and fixed in ice-cold 70% ethanol for 24 h at -20°C. After fixation, the cells were washed twice with EBSS, and DNA was extracted by 5 min of incubation with the DNA extraction buffer (200 mM Na_2_HPO_4_ with 0.004% Triton X-100, pH 7.8). Then the cells were washed with EBSS, resuspended in the DNA staining solution (EBSS, 50 mg/ml propidium iodide, 0.2 mg/mL DNAse-free RNAse A), and analyzed using the Attune NxT flow cytometer (Life Technologies). The data were analyzed using the Attune NxT flow cytometer Software (Life Technologies).

### Analysis of Phosphatidylserine Externalization

To investigate apoptosis in A549 and Lewis cells, we used Annexin V for detection of phosphatidylserine externalization—one of the early apoptosis markers. Briefly, cells were seeded in six-well culture plates (2 × 10^5^ cells per well) at pH 5.5, grown for 24 h, and incubated with 1 μM mambalgin-2 for 72 h without the media change. After incubation, the cells were detached using the Versene solution and washed with annexin-binding buffer (V13246, Thermo Fisher Scientific). Then, the cells were incubated with Annexin V conjugated to Alexa 488 (A13201, Thermo Fisher Scientific) for 20 min, washed with the annexin-binding buffer, and analyzed using the Attune NxT flow cytometer (Life Technologies). The data were analyzed using the Attune NxT Software (Life Technologies).

### Patch-Clamp Recordings

Electrophysiological experiments on different cell lines (A549, WI-38, and HLF) were performed using the whole-cell configuration of the patch-clamp technique. Cells were placed (passaged) on sterile 4 × 4-mm coverslips in 35-mm Petri dishes 1–2 days before the experiment. Ion current records were acquired with the Axopatch 200B amplifier (Molecular Devices, San Jose, CA, USA), the analog–digital interface Digidata 1550A and PC running the Clampex software (Molecular Devices). Borosilicate micropipettes (BF‐150‐110‐10, Sutter Instruments, Novato, CA, USA) were pulled at a P-97 puller (Sutter Instrument) to a resistance of 3–6 MΩ when filled with a relevant intracellular solution containing (in mM) 140 K-Aspartate, 5 NaCl, 1 MgCl_2_, 2 EGTA/KOH, 20 HEPES/TRIS, and 0.176 CaCl_2_ to establish free ionized calcium concentration [Ca^2+^]i at 0.01 µM. Typical bath solution (in the chamber) contained (in mM) 145 NaCl, 2 CaCl_2_, 1 MgCl_2_, and 10 HEPES/TRIS (pH 7.4). For bath extracellular solution with pH 5.5, HEPES/TRIS was replaced by MES. Gap-free ion currents were recorded at holding membrane potential -50 mV. Data were low-pass filtered at 200 Hz and analyzed using Axon pClamp 10.5 software suite (Molecular Devices).

### Immunoprecipitation, Affinity Purification, and Western Blotting

For investigation of the mambalgin-2 targets in A549 cells, mambalgin-2 (1 mg/ml) was coupled to NHS-activated Sepharose 4 Fast Flow (Cat #17-0906-01, GE Healthcare, Chicago, IL, USA) according to the manufacturer’s instructions. The resin blocked by 500 mM ethanolamine without any protein coupled was used as a negative control. The membrane fraction of A549 cells (5 × 10^7^ cells per sample) was solubilized in 2% Triton X-100 (Cat# A4975, Panreac, Barcelona, Spain), diluted 10 times with TBS buffer (100 mM TRIS, 150 mM NaCl, pH 8.0), and incubated with the resin for 16 h at 4°C in TBS. After that, non-specifically bound proteins were sequentially washed out from the resin with five volumes of TBS, five volumes of TBS + 1 M NaCl + 0.5% Triton X-100, and five volumes of TBS + 0.5% Triton X-100. The specifically bound proteins were eluted by five volumes of 200 mM glycine (pH 2.6), diluted in the loading buffer (120 mM Tris–HCl, 20% [v/v] glycerol, 10% [v/v] mercaptoethanol, 4% [w/v] sodium dodecyl sulfate, and 0.05% [w/v] bromophenol blue, pH 6.8), submitted to gel electrophoresis, blotted onto nitrocellulose membranes (GE Healthcare), and blocked for 2 h in 5% skim milk (Sigma-Aldrich) in TBS (50 mM Tris, 150 mM NaCl, pH 7.4) buffer + 0.1% Tween-20 (AppliChem, Darmstadt, Germany). The membranes were then incubated overnight at 4°C with primary antibodies against ASIC1a (mouse, 1:1,000, SMC-427, StressMarq Biosciences, Victoria, Canada), α-ENaC (rabbit, 1:1,000, ABIN1841945, Antibodies-Online), or γ-ENaC (mouse, 1:1,000, ABIN1865926, Antibodies-Online), washed three times with TBS + 0.1% Tween-20, and incubated with HRP-conjugated secondary anti-rabbit antibody (1:5,000, 111-035-003, Jackson ImmunoResearch), in the case of α-ENaC or anti-mouse antibody (1:5,000, 715-035-150, Jackson ImmunoResearch) in the cases of ASIC1a or γ-ENaC for 1 h (20°C). After that, membranes were washed four times with TBS + 0.1% Tween-20, and an HRP signal was detected by ECL substrate (Bio-Rad) using the ImageQuant LAS 500 chemidocumenter (GE Healthcare).

To study the formation of the ASIC1/α-ENaC/γ-ENaC heterotrimers in A549 cells, 100 µg of ASIC1a antibody (sheep, final concentration 1 mg/ml ABIN350049, Antibodies-Online) was conjugated to Protein-A agarose (Cat #10037259, Roche) in accordance with the manufacturer’s protocols. The membrane fraction of A549 cells (5 × 10^7^ cells per sample) was solubilized in 2% Triton X-100 (Panreac) and diluted 10 times with TBS buffer (100 mM Tris, 150 mM NaCl, pH 8.0). After that, the lysates were pre-cleared by empty Protein A-conjugated agarose (Roche) for 4 h at RT to avoid non-specific binding and then incubated with the ASIC1a antibody-coupled agarose for 16 h at 4°C in TBS. The empty agarose blocked with 1% skim dry milk (Sigma-Aldrich) was used as a negative control. After that, non-specifically bound proteins were sequentially washed out from the resin with five volumes of TBS, five volumes of TBS + 1 M NaCl + 0.5% Triton X-100, and five volumes of TBS + 0.5% Triton X-100. The specifically bound proteins were eluted by 10 min of heating in the loading buffer (120 mM Tris–HCl, 20% [v/v] glycerol, 10% [v/v] mercaptoethanol, 4% [w/v] sodium dodecyl sulfate, and 0.05% [w/v] bromophenol blue, pH 6.8), submitted to gel electrophoresis, blotted onto nitrocellulose membranes (GE Healthcare), and blocked for 2 h with 5% skim milk (Sigma-Aldrich) in TBS buffer (50 mM Tris, 150 mM NaCl, pH 7.4) + 0.1% Tween-20. The membranes were incubated overnight at 4°C with the primary antibodies against α-ENaC (rabbit, 1:1,000, ABIN1841945, Antibodies-Online) or γ-ENaC (mouse, 1:1,000, ABIN1865926, Antibodies-Online), washed three times with TBS + 0.1% Tween-20, and incubated with the HRP-conjugated secondary anti-rabbit antibody (1:5,000, 111-035-003, Jackson ImmunoResearch), in the case of α-ENaC or anti-mouse antibody (1:5,000, 715-035-150, Jackson ImmunoResearch) in the case of γ-ENaC for 1 h (20°C). After that, the membranes were washed four times with TBS + 0.1% Tween-20, and an HRP signal was detected by ECL substrate (Bio-Rad) using the ImageQuant LAS 500 chemidocumenter (GE Healthcare).

### Expression of ASICs/ENaCs in *X. laevis* Oocytes

For expression of the human homomeric ASIC1a (hASIC1a) and human heteromeric ASIC1a/α-ENAC/γ-ENAC (hASIC1a/α-ENAC/γ-ENAC) channels in *Xenopus laevis* oocytes, the linearized plasmids were transcribed using the T7 mMESSAGE-mMACHINE Transcription Kit (Thermo Fischer Scientific). Oocytes were defolliculated and injected with 2.5–10 ng of mRNA. mRNA transcripts of hASIC1a, hENACα, and hENACγ were synthesized by the mMESSAGE-mMACHINE T7 kit (Cat# AM1344, Thermo Fisher Scientific) according to the protocol for capped transcripts supplied by the manufacturer. For hASIC1a/ENACα/ENACγ expression, the corresponding mRNAs were mixed at the 1:1:1 molar ratio. The preparation of *Xenopus* oocytes at defolliculated stages V–VI was done as previously described (29). Non-injected defolliculated oocytes were used as the control for an absence of endogenous ASIC expression. After injection, the oocytes were kept for 2–3 days at 19°C and then up to 7 days at 15°C in ND-96 medium supplemented with gentamycin (Cat# G1264, Merck, Darmstadt, Germany) (50 μg/ml) and containing (in mM) 96 NaCl, 2 KCl, 1.8 CaCl_2_, 1 MgCl_2_, and 10 HEPES, pH 7.4. Plasmids bearing hASIC1a, hα-ENAC, and hγ-ENAC were kindly provided by Dr. Alexander Staruschenko (University of South Florida, Tampa, FL, USA).

### Electrophysiological Recordings in *X. laevis* Oocytes

Two-electrode voltage-clamp recordings were performed using the GeneClamp500 amplifier (Axon Instruments, Inverurie, UK) at the holding potential of -50 mV as described previously (29). The output signal was filtered at 20 Hz and digitized at 100 Hz using the L780 AD converter (L-Card, Moscow, Russia). Microelectrodes were filled with 3 M KCl. The external perfusion solution of oocytes was ND-96 with pH 7.4 for both hASIC1a and hASIC1a/α-ENAC/γ-ENAC, and it was fed to a perfusion chamber (volume ~50 µl) by a gravity-flow system at 2.5 ml/min. The currents were stimulated by a pH drop to 5.0 for both hASIC1a and hASIC1a/α-ENAC/γ-ENAC.

The pH drop was done by fast perfusion solution exchange for 0.1 s to ND-96 containing 10 mM MES instead of HEPES and adjusted to the target pH. The pH drop was kept for 7 s, and the solution was exchanged to ND-96 with pH 7.4 afterward. The treatment of oocytes with the mambalgin-2-containing solution was performed by solution exchange in the recording chamber for 15 s before the current stimulation. Lyophilized mambalgin-2 was dissolved in 100% DMSO to a 10-mM stock solution and then diluted to a target concentration with ND-96 right before the experiment. Amiloride (Sigma-Aldrich) was diluted in ND-96 (pH 7.4) from the 500-mM stock in 100% DMSO. The concentration of DMSO in the recording solution was kept below 1% in the all experiments. The exchange of extracellular solutions was performed using a computer-controlled valve system.

The dose–response curves for mambalgin-2 were recorded in the 0–10-μM concentration range. Each oocyte was tested with all concentrations of mambalgin-2. Dose–response curves were fitted using the Hill equation: Y = 100%/(1 + 10^((LogIC_50_ - X)*nH)), where Y is the normalized current amplitude (the normalization was done independently for each oocyte), X is the ligand concentration in the logarithmic scale, and nH is the Hill coefficient (slope factor).

### TCGA Database Analysis

Data of the TCGA GTEX (healthy lung biopsies) and LUAD (lung adenocarcinoma) studies were accessed *via* the UCSC Xena platform (30). For comparison of the ASIC and ENaC expressions in normal lungs, adenocarcinomas, and adenocarcinoma-surrounding tissues, the data on the mRNA expression of the genes *ACCN2*, *ACCN1*, *ACCN3*, *ACCN4*, *SCNN1A*, and *SCNN1G* coding the ASIC1, ASIC2, ASIC3, ASIC4, α-ENaC, and γ-ENaC subunits, respectively, were downloaded from the GTEX and LUAD studies and analyzed by the GraphPad Prism 8.0 software (GraphPad Software). Please note that the genes coding the ASIC1 and ASIC2 subunits are designated as *ACCN2* and *ACCN1*, respectively. For analysis of the relationships between the ASIC/ENaC mRNA expression and lung adenocarcinoma patients’ survival, the patients with lung adenocarcinoma on the I stage and on the II–IV stages were subdivided into two groups with the *ACCN2*, *ACCN1*, *ACCN3*, *ACCN4*, *SCNN1A*, and *SCNN1G* expression above (high) or below (low) the median value. Overall survival curves were analyzed according to the Kaplan–Meier method for estimation of the survival function from the lifetime data, and lifetime functions were compared using the log-rank test directly in the UCSC Xena platform interface.

### Statistical Analysis

Data are presented as mean ± SEM. Sample numbers (n) are indicated in the figure legends. No exclusion criteria were applied for the experimental data. The absence of the outliers in each dataset was confirmed by the Grubbs’ test (alpha = 0.05). Before the comparisons, the data were tested for normality (Shapiro–Wilk test, at p = 0.05) and for the homogeneity of variances (Levene’s test, at p = 0.05). The data were analyzed using the one-way ANOVA with appropriate multiple-comparison *post-hoc* test, one-sample t-test, two-tailed t-test for normally-distributed data, and Welch’s unequal variance t-test for data without the variance homogeneity as indicated in the figure legends. For comparison of the patients’ survival with the different mRNA expressions of ASICs and ENaCs, the log-rank test was used. Differences in the data were considered statistically significant at p < 0.05. Analysis was performed using the GraphPad Prism 8.0 software (GraphPad Software).

## Results

### Mambalgin-2 Inhibits the Growth and Migration of Lung Adenocarcinoma Cells

Previously, we showed that the recombinant analogue of mambalgin-2 inhibits the growth of leukemia (15) and glioma cells (16) and the growth and migration of melanoma cells (17). Here, we tested mambalgin-2’s effect on lung adenocarcinoma A549 and Lewis cells, lung transformed WI-38 fibroblasts, and lung normal HLF fibroblasts. As the acidification of the cell media results in stimulation of the growth, migration, and invasion of melanoma cells and influences the expression pattern of the ASIC and ENac subunits (17), we studied mambalgin-2’s activity in cells cultivated in the normal (pH 7.4) and acidic (pH 5.5) media.

**Figure 1:**
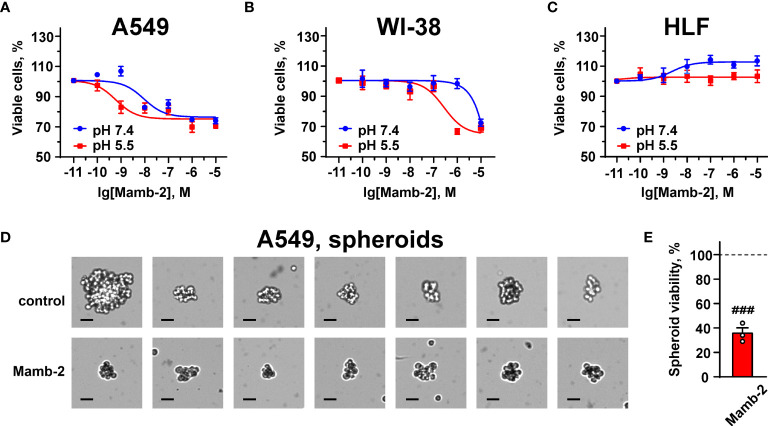
Dose–response effects of mambalgin-2 on viability of A549 **(A, D, E)**, WI-38 **(B)**, and HLF **(C)** cells. **(A–C)** Cells were cultured at pH 7.4 and at pH 5.5. Data are presented as % of the control (untreated cells) ± SEM (n = 4). **(D)** Examples of the spheroids reconstructed from A549 cells incubated in the absence or presence of 1 µM of mambalgin-2 after staining by Trypan Blue, scale bar = 25 µm. **(E)** Influence of 1 µM of mambalgin-2 on the viability of the spheroids reconstructed from A549 cells. Data are presented as % of the control (untreated spheroids) ± SEM (n = 3). ^###^(p < 0.001) indicates difference between untreated and treated spheroids according to one-sample t-test.

WST-1 assay revealed that mambalgin-2 reduced the proliferation of A549 cells cultivated in both the normal and acidic media up to ~75% of the control (untreated cells) upon 72 h of incubation with the toxin. Mambalgin-2 demonstrated a dose-dependent activity with EC_50_ 9.29 ± 0.9 nM and 0.59 ± 0.1 nM in A549 cells cultivated at pH 7.4 and 5.5, respectively (**Figure 1A**). The maximal antiproliferative effect of mambalgin-2 on A549 cells was observed at the 1 µM concentration (**Figure 1A**). Mambalgin-2 did not affect the growth of Lewis lung adenocarcinoma (Lewis cells) at any pH but inhibited the growth of Lewis metastatic subline P29 (Lewis-P29 cells) at pH 5.5 with EC_50_ 17.47 ± 5.3 µM (**Figures S2A, B**).

Mambalgin-2 did not affect the viability of WI-38 fibroblasts cultivated in the normal media up to the 1 µM concentration but suppressed their growth up to ~65% upon cultivation in the acidic medium with EC_50_ significantly higher than that found for A549 cells (276.5 ± 14.3 nM, **Figure 1B**). Notably, no significant effect of mambalgin-2 on the growth of HLF fibroblasts cultivated at either pH 7.4 or 5.5 was observed (**Figure 1C**).

**Figure 2:**
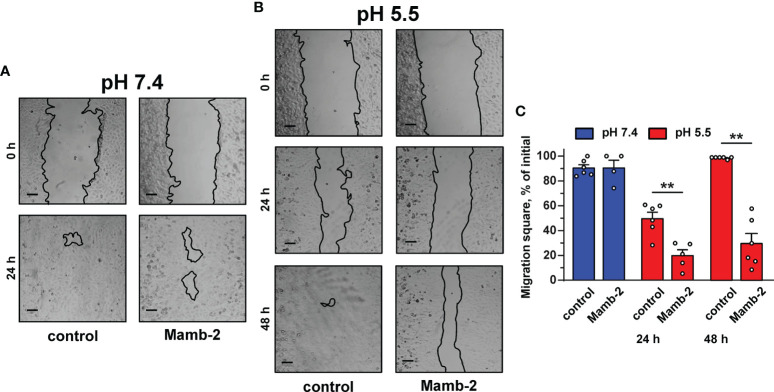
Influence of mambalgin-2 on migration of A549 cells cultivated at pH 7.4 and 5.5. **(A, B)** Representative pictures of wounds for A549 cells incubated at pH 7.4 **(A)** and at pH 5.5 **(B)** in the absence or presence of 1 µM of mambalgin-2. Cells were incubated with mambalgin-2 at pH 7.4 for 24 h and at pH 5.5 for 48 h. Scale bar = 100 µm. **(C)** Wound area occupied by migrating A549 cells. Data are presented as % of the wound surface, occupied by migrating cells ± SEM (n = 6), **(p < 0.01) indicates significant difference between the data groups by two-tailed t-test.

Study of 1 µM mambalgin-2’s influence on the migration of A549 cells by wound healing assay revealed the toxin’s inhibitory activity only at acidic conditions (**Figure 2**). Incubation with mambalgin-2 for 24 and 48 h at pH 5.5 resulted in 2- and 3-fold decreases in the cell motility, respectively (**Figures 2B, C**). Similarly, 1 µM mambalgin-2 affected the migration of Lewis-P29 cells only at pH 5.5 (**Figure S3**). Thus, we can conclude that mambalgin-2’s effect on A549 cells observed by wound healing assay is most likely related with influence on the cell migration rather than on their growth, while the toxin action on the growth and migration of Lewis-P29 cells was equal.

Media acidification per se significantly inhibited the migration of A549, WI-38, and HLF cells upon 24 h of incubation with the most prominent effect in HLF cells even in the absence of mambalgin-2 (**Figures S4A–F**). The inhibition effect of acidification was increased in the series A549 < WI-38 < HLF. However, incubation of A549 cells upon 48 h at acidic conditions restored the cell migratory activity (**Figure 2B**), pointing on the successful adaptation of the cells to the acidic media. The media acidification did not influence the viability of A549 and WI-38 cells, while it significantly reduced the proliferation of HLF cells (**Figure S4G**). In contrast, mamblagin-2 inhibited the growth of A549 and WI-38 cells, and no effect was found in normal fibroblasts (**Figure 1**). Thus, we can conclude that the effects observed in the studied cells in the presence of mambalgin-2 are conditioned by the toxin’s interaction with its targets.

To translate mambalgin-2’s effects on a 3D cell model, we used multicellular spheroids reconstructed from A549 cells. Analysis with Trypan Blue showed that the control spheroids (in the absence of mambalgin-2) were composed of viable cells as they were impermeable for Trypan Blue. However, the spheroids incubated with 1 µM mambalgin-2 for 24 h became permeable for Trypan Blue (**Figure 1D**). This points on the loss of cell membrane integrity of the spheroids upon mambalgin-2 treatment. WST-1 assay confirmed that mambalgin-2 inhibited the viability of cells in the spheroids up to ~37% from the control level (untreated spheroids, **Figure 1E**).

### Mambalgin-2 Induces G2/M Cell Cycle Arrest and Apoptosis in Lung Adenocarcinoma Cells

**Figure 3:**
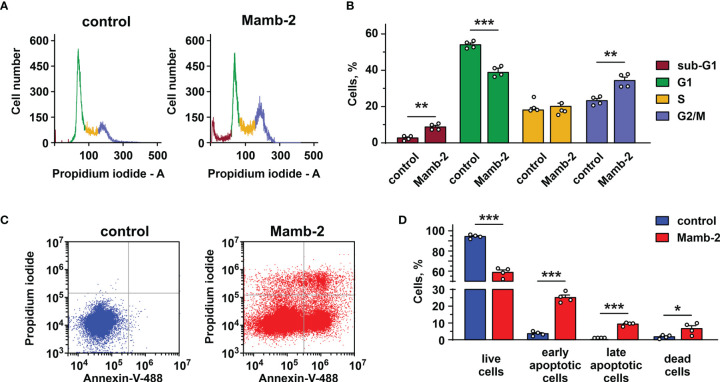
Analysis of cell cycle and apoptosis induction by mambalgin-2 in A549 cells. **(A)** Representative nucleus population distribution of A549 cells incubated in the absence (control) or presence of 1 µM mambalgin-2. **(B)** % of cells in each cell cycle phase. Data are presented as % of cells in each cell cycle phase ± SEM (n = 4). ** (p < 0.01) and *** (p < 0.001) indicate significant difference between the data groups by two-tailed t-test. **(C)** Representative pictures of phosphatidylserine externalization analysis upon the 1 µM mambalgin-2 treatment of A549 cells by flow cytometry with Annexin V-488 and propidium iodide (control is in absence of mambalgin-2). **(D)** Percentage of A549 cells with externalized phosphatidylserine and bound propidium iodide in the absence (control) or presence of 1 µM mambalgin-2. The data are presented as % of live, early apoptotic, late apoptotic, and dead cells ± SEM (n = 4). * (p < 0.05) and *** (p < 0.001) indicate the significant difference between data groups by a two-tailed t-test.

Previously, we showed that mambalgin-2 induces the cell cycle arrest and apoptosis in leukemia and glioma cells (15, 16). Here, we analyzed the toxin’s action in A549 and Lewis-P29 cells at pH 5.5. Flow cytometry analysis revealed that in A549 cells, mambalgin-2 reduces the cell number in the G1 phase from ~54% to ~38% and increases the cell number in the G2/M phase from ~23% to ~34% (**Figures 3A, B**), pointing on the cell cycle arrest in the G2/M phase. A significant increase in the sub-G1 population (from ~2.7% to 8.8%, **Figure 3B**) is characteristic for apoptosis (31). Indeed, the analysis by flow cytometry revealed that the number of A549 cells with externalized phosphatidylserine significantly increased upon the incubation with 1 μM mambalgin-2 from ~3.6% to ~25.2% (**Figures 3C, D**). Moreover, ~9.4% of A549 cells possessed not only externalized phosphatidylserine but also bound propidium iodide upon mambalgin-2 treatment (**Figures 3C, D**). This points to the membrane integrity loss and the late apoptosis induction. An even more profound G2/M cell cycle arrest was observed in Lewis-P29 cells upon incubation with 1 μM mambalgin-2 (**Figures S5A, B**), although the apoptosis induction was not so prominent as in the case of A549 cells (~ 12% of early apoptotic cells) (**Figures S5C, D**).

### Expression of mRNAs Coding ASIC and ENaC Subunits in Lung Transformed and Normal Cells

**Figure 4:**
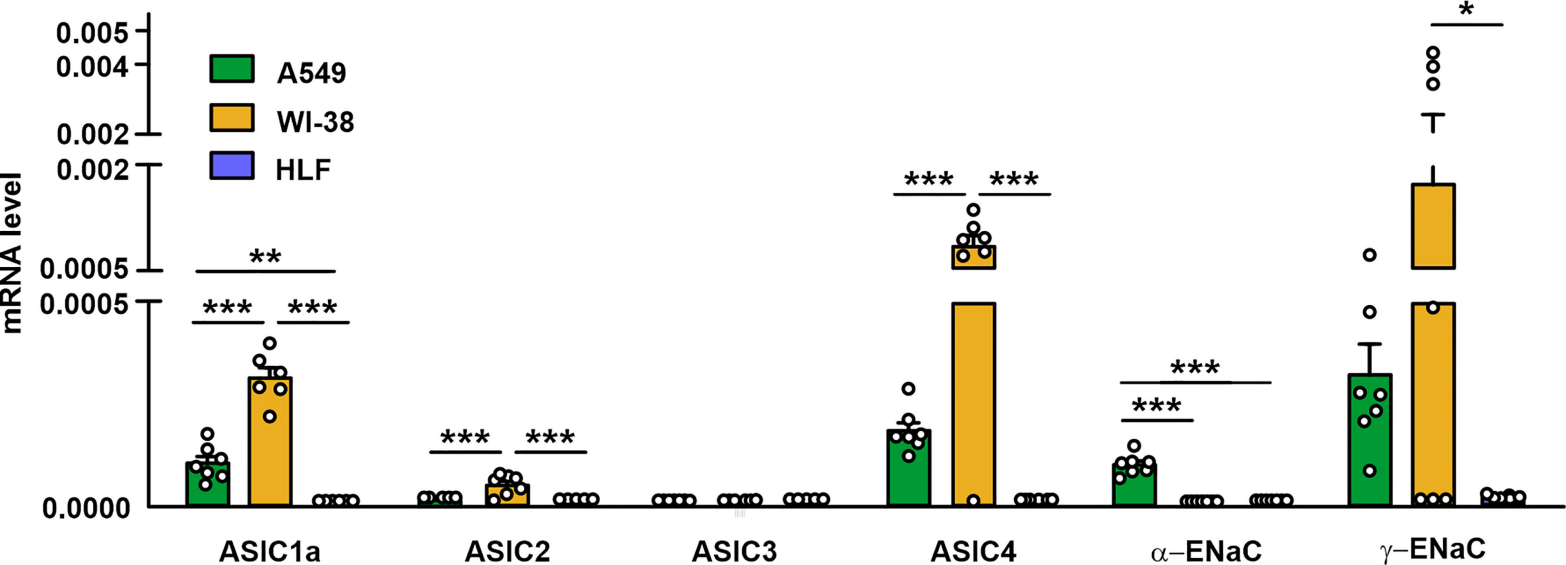
qPCR analysis of the *ACCN2*, *ACCN1*, *ACCN3*, *ACCN4*, *SCNN1A*, and *SCNN1G* expression in A549, WI-38, and HLF cells. Gene expression was normalized to the *β-ACTIN*, *GPDH*, and *RPL13a* housekeeping genes and presented as relative mRNA level ± SEM (n = 6). * (p < 0.05), ** (p < 0.01), and *** (p < 0.001) indicate significant difference between the data groups according to one-way ANOVA followed Tukey’s *post-hoc* test, n.s. - no significant difference (p>0.05).

As mambalgin-2’s action in A549 and Lewis-P29 cells was pH-dependent (**Figures 1A**, **2C**, **S2B**, **S3C**), we proposed that it can be connected with the expression of some ASIC and/or ENaC subunits. In order to elucidate the expression pattern of the DEG/ENaC channels and to explain the reason of the mambalgin-2 selectivity in different lung-derived cells, we analyzed the expression of the *ACCN2*, *ACCN1*, *ACCN3*, *ACCN4*, *SCNN1A*, and *SCNN1G* genes coding the ASIC1a isoform, ASIC2, ASIC3, ASIC4, α-ENaC, and γ-ENaC subunits, respectively, in A549, Lewis, WI-38, and HLF cells by real-time PCR. We found that mRNAs of all the investigated channels except ASIC3 were presented in A549 cells. WI-38 fibroblasts demonstrated a higher expression of mRNA coding the ASIC1a, ASIC2, ASIC4, and γ-ENaC subunits than in A549 cells. In contrast, the expression of mRNA coding α-ENaC was significantly down-regulated in WI-38 cells in comparison with A549 cells (**Figure 4**). Surprisingly, HLF cells demonstrated little expression only of mRNA coding γ-ENaC, while the expression of the genes coding other ASIC and ENaC subunits was not found at all (**Figure 4**). Parental Lewis cells did not express ASIC1a at all and demonstrated the significantly lower expression of α-ENaC than metastatic Lewis-P29 cells, while all the studied ASIC and ENaC genes were presented in Lewis-P29 cells (**Figure S6**). As mambalgin-2 did not affect the proliferation of parental Lewis cells (**Figure S2**), we propose that the toxin’s influence on the lung cancer cell growth is related at least with the ASIC1a expression.

### Functional Expression of DEG/ENaC Channels in Lung Adenocarcinoma Cells and Immortalized Lung Fibroblasts

**Figure 5:**
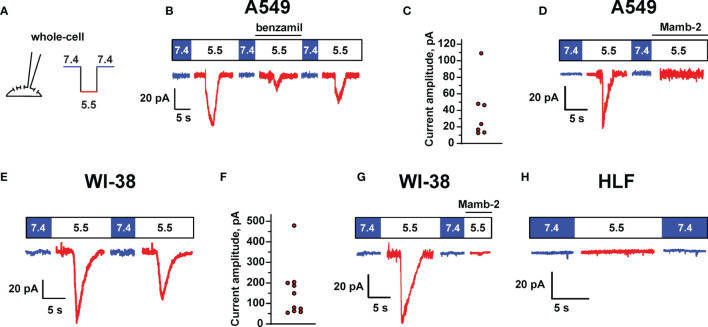
ASIC channel activity in A549, WI-38, and HLF cells. **(A)** Whole-cell configuration of the patch clamp technique and protocol of experiments. Membrane voltage was clamped at –50 mV in all patches. **(B)** Representative traces showing ASIC1a-like currents activated by a rapid drop of extracellular pH from 7.4 to 5.5, the currents were reversibly inhibited by 10 μM benzamil (derivative of amiloride). **(C)** Distribution of transient peak amplitudes at pH 5.5 elicited ASIC1a-like currents in A549 cells. **(D)** The effect of mambalgin-2 on ASIC1a-like currents in A549 cells. **(E)** ASIC1a-like currents in WI-38 cells. **(F)** Distribution of transient peak amplitudes in WI-38 cells at pH 5.5 elicited ASIC1a-like currents. **(G)** The effect of mambalgin-2 on ASIC1a-like currents in WI-38 cells. **(H)** An absence of acidification-evoked currents in HLF cells.

To determine the presence of the functionally active ASIC-like channels in lung cancer and normal cells, electrophysiological experiments in A549, WI-38, and HLF cells were carried out using the patch-clamp technique in the whole-cell configuration (**Figure 5A**). It was found that a rapid change in the pH value of the external (bath) solution from 7.4 to 5.5 in most cases activated the inward transmembrane currents in A549 cells with a peak amplitude from 12.8 to 109 pA (**Figures 5B, C**). A decrease in pH led to the rapid increase in the current amplitude to a maximum followed by the slower decrease in the course of desensitization to a stationary level, which is typical for the acid-sensing ion channel ASIC1a. The addition of 10 µM benzamil [analogue of amiloride with IC_50_ ~ 3.50 µM at the ASIC1 channels (24)] to the extracellular solution caused the suppression of the inward currents activated by acidification of the extracellular solution to pH 5.5 up to ~25% of the initial value (**Figure 5B**). Subsequent replacement of the extracellular solution with the solutions without benzamil (with pH 7.4 and 5.5) led to the reactivation of the ASIC channels (**Figure 5B**). The data obtained indicate the expression of the functionally active ASIC1a channels in the membrane of A549 cells. Similar to A549 cells, in the whole-cell experiments in immortalized WI-38 fibroblasts, we also recorded the currents induced by the rapid drop in the pH value of the extracellular solution from 7.4 to pH 5.5 with the kinetic parameters, which are characteristic for the ASIC1a-containing channels. The amplitude of the currents was in the range from 54.7 to 479.1 pA (**Figures 5E, F**). In line with nearly zero expression of the ASIC genes (**Figure 4**) and absence of mambalgin-2’s effect on HLF cells (**Figure 1C**), the whole-cell experiments in lung primary fibroblasts did not reveal the presence of any acidification-induced currents (**Figure 5H**).

Finally, we studied the effect of 1 μM mambalgin-2 on the acid-sensing ion currents in A549 and WI-38 cells. The addition of mambalgin-2 to the extracellular solution fully inhibited the channel activity at pH 5.5 in both types of cells (**Figures 5D, G**). Thus, mambalgin-2 selectively inhibits the growth of the cells, which express the functional ASIC1a containing channels on their plasma membrane.

### Mambalgin-2 Binds to ASIC1a/α-ENaC/γ-ENaC Heterotrimer in Lung Adenocarcinoma Cells

Different mambalgin-2 effects (**Figures 1A, B**) and expression patterns of the genes coding the ASIC and ENaC subunits (**Figure 4**) in A549 and WI-38 cells allowed us to hypothesize that mambalgin-2 has another acid-sensitive target in cancer cells than the described earlier homomeric ASIC1a channels (26).

**Figure 6:**
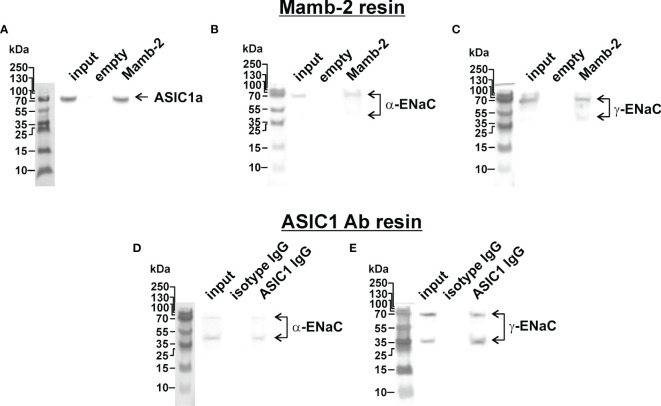
Investigation of mambalgin-2 interaction with ASIC1, α-ENaC, and γ-ENaC subunits in A549 cells. Western blot analysis of the ASIC1 **(A)**, α-ENaC **(B)**, and γ-ENaC **(C)** subunits extraction from the membrane fraction of A549 cells by affinity chromatography using NHS-Sepharose resin coupled with mambalgin-2 (n = 4). Whole Western blotting membranes are shown in **Figure S7**. Western blot analysis of the α-ENaC **(D)** and γ-ENaC **(E)** subunits extracted from the membrane fraction of A549 cells after its precipitation using Protein A-agarose conjugated with ASIC1-antibody (n = 4). Whole Western blotting membranes are shown in **Figure S8**.

Recently, we have proposed that the target of mambalgin-2 in melanoma cells could be the heteromeric acid-sensitive channels ASIC1/α-ENaC/γ-ENaC (17). To investigate whether mambalgin-2 interacts with the ASIC1a, α-ENaC, and γ-ENaC subunits in A549 cells, we performed an extraction of these subunits from the membrane fraction of A549 cells by affinity chromatography using an N-hydroxysuccinimide resin coupled with mambalgin-2. The empty resin blocked by 500 mM ethanolamine was used as a negative control. Analysis by Western blotting revealed that mambalgin-2 extracted detectable amounts of the ASIC1a, α-ENaC, and γ-ENaC subunits from the membrane fraction of A549 cells (**Figures 6A–C** and **S7**). To confirm the formation of the ASIC1a/α-ENaC/γ-ENaC heterotrimers, we performed the immunoprecipitation of the ASIC1a partners from the membrane fraction of A549 cells using the protein A-agarose coupled with the antibody against ASIC1a. Western blotting revealed extraction of the α-ENaC and γ-ENaC subunits (**Figures 6D, E** and **S8**) pointing on the direct interaction of ASIC1a with α-ENaC and γ-ENaC. Altogether, our data point on the formation of the ASIC1a/α-ENaC/γ-ENaC heterotrimers in A549 cells and on the possible interaction of mambalgin-2 with these heteromeric channels.

### Mambalgin-2 Inhibits ASIC1a/α-ENaC/γ-ENaC Heterotrimer Much More Effectively Than ASIC1a Homotrimer

**Figure 7:**
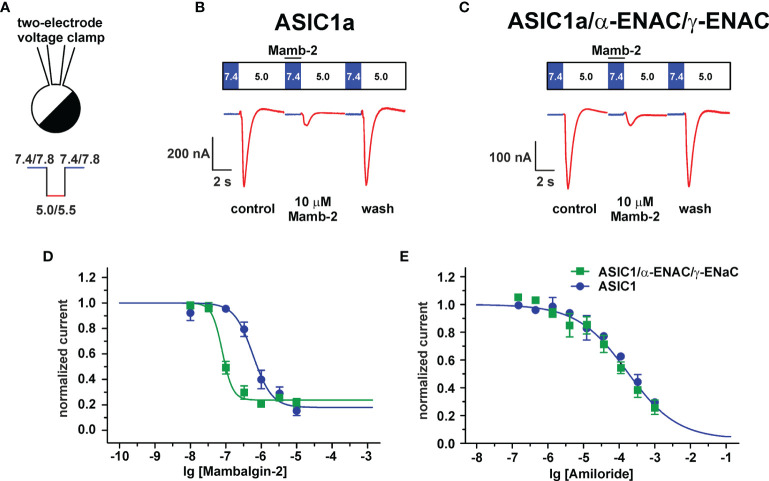
Analysis of mambalgin-2 and amiloride action at ASIC1a and ASIC1a/α-ENaC/γ-ENaC channels in *Xenopus laevis* oocytes. **(A)** Two-electrode configuration of the patch-clamp technique and protocol of experiments. Representative current traces recorded in *X. laevis* oocytes expressing the hASIC1a **(B)** and hASIC1a/α-ENAC/γ-ENAC **(C)** channels; traces for non-injected oocytes were used as control and are shown in **Figure S9**. The pre-incubation with mambalgin-2 was 15 s (shown off time scale), the stimulation phase (pH 5.0) was 7 s, and the recovery phase is not shown. **(D)** Dose–response curves for mambalgin-2 inhibitory effect. For each mambalgin-2 concentration, the current response was normalized to the control experiment. Each data point represents an average from independent experiments in different oocytes ± SEM (n =11 for both channels). The fitted curves are described by single-component Hill’s equation. **(E)** Dose–response curves for amiloride inhibitory effect. The data points are shown as normalized average ± SEM (n = 3 for both channels).

To finally prove that the ASIC1a/α-ENaC/γ-ENaC heterotrimers can be the target of mambalgin-2, we studied the toxin’s ability to modulate the acidification-induced currents in *X. laevis* oocytes expressing the human ASIC1a, α-ENAC, and γ-ENAC subunits in a ratio 1:1:1 using the two-electrode voltage clamp technique (**Figure 7A**). We have observed the current responses stimulated by the pH drop from 7.4 to 5.0, which could be reversibly inhibited by the mambalgin-2 treatment before the stimulation (**Figures 7B, C**). No currents were elicited by the pH drop in non-injected oocytes, confirming that the observed responses could be attributed only to heterologous expression (**Figure S9**). Surprisingly, mambalgin-2 inhibited the heterotrimeric hASIC1a/α-ENAC/γ-ENAC channels with significantly higher efficacy than the hASIC1a homotrimeric channels (**Figure 7D**). The inhibitory action of mambalgin-2 on both receptor types is described by the single-component Hill’s equation, characterized by the following parameters: IC_50_ = 79 ± 5 nM, n_H_ = 2.9 ± 0.5, bottom = 0.23 ± 0.02 and IC_50_ = 0.59 ± 0.13 μM, n_H_ = 1.6 ± 0.5, bottom = 0.18 ± 0.06 for hASIC1a/α-ENAC/γ-ENAC and hASIC1a, respectively. Notably, the dose–response curves for amiloride were identical at the hASIC1a and hASIC1a/α-ENAC/γ-ENAC channels (F-test F m 3, 32 = 1.009; **Figure 7E**). Thus, we confirmed that mambalgin-2 is an inhibitor of the ASIC1a/α-ENaC/γ-ENaC heterotrimeric channels.

### Anti-Proliferative and Anti-Migratory Activity of Mambalgin-2 at Low pH Is Dependent on the Expression of ASIC1a, α-ENaC, and γ-ENaC

**Figure 8:**
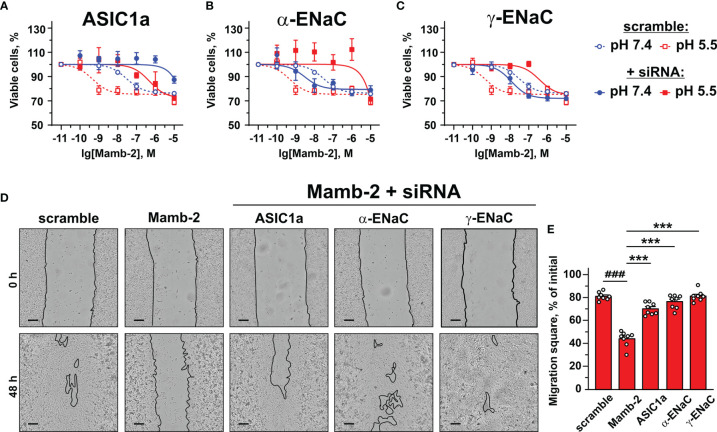
Influence of knockdown of ASIC1, α-ENaC, and γ-ENaC expression on mambalgin-2 activity in A549 lung cancer cells. Dose–response effects of mambalgin-2 on viability of A549 cells after cell transfection with scramble siRNA or siRNA specific for ASIC1a **(A)**, α-ENaC **(B)**, and γ-ENaC. **(C)** Data are presented as % of the control (untreated cells) ± SEM (n = 4). The gene knockdown confirmation is shown in **Figures S1A–E**, and the analysis of dose–response curves is given in **Table 1**. **(D)** Representative pictures of wounds for A549 cells incubated at pH 5.5 in the presence of 1 µM of mambalgin-2 upon knockdown of ASIC1a, α-ENaC, and γ-ENaC genes. Scale bar = 100 µm. Analysis of gene knockdown influence on the migration of A549 cells in the absence of mambalgin-2 is given in **Figures S1F, G**. **(E)** Wound area occupied by migrating A549 cells. Data are presented as % of the wound surface, occupied by migrating cells ± SEM (n = 8); ^###^(p < 0.001) and *** (p < 0.001) indicate significant difference between the data groups by one-way ANOVA followed by Tukey’s *post-hoc* test.

In order to confirm the implication of the members of DEG/ENac superfamily in the anti-proliferative activity of mambalgin-2, we performed the knockdown of the genes coding the ASIC1a, α-ENaC, and γ-ENaC subunits by siRNA in A549 cells. ASIC1 knockdown completely abolished the mambalgin-2 activity at pH 7.4 and significantly decreased it at pH 5.5 (**Figure 8A**, **Table 1**). However, the down-regulation of the α-ENaC and γ-ENaC expression significantly decreased the mambalgin-2 activity only at pH 5.5 and even slightly increased it at pH 7.4 (**Figures 8B, C**). These results mean that mambalgin-2 can regulate the growth of A549 cells even at neutral pH, and this effect is mediated by ASIC1. Notably, the ASIC1a, α-ENaC, and γ-ENaC knockdown did not affect the A549 cell growth per se both at 7.4 and 5.5 pH (**Figure S1E**).

**Table 1:** Parameters describing the effect of ASIC1a, α-ENaC, and γ-ENaC knockdown on the anti-proliferative activity of mambalgin-2 in A549 cells.

Gene knockdown	pH 7.4		pH 5.5	
	A_1_, %	EC_50_, nM	A_1_, %	EC_50_, nM
Scramble	76.63 ± 1.2	26 ± 1.9	75.42 ± 1.7	0.4 ± 0.02
ASIC1a	n.d.	n.d.	72.35 ± 6.5	507.4 ± 27.7*^a^*
α-ENaC	79.44 ± 2.7	3.5 ± 0.4	n.d.	n.d.
γ-ENaC	71.61 ± 1.8	14.15 ± 2.36	74.69 ± 2.6	375.3 ± 25.3*^a^*

^a^Significant difference between EC_50_ values of mambalgin-2 upon and without knockdown of the corresponding gene according to F-test (p < 0.001, n = 4).

The migration assay revealed that knockdown of the α-ENaC expression impaired the migration of A549 cells at pH 5.5, while knockdown of the ASIC1a and γ-ENaC expression did not (**Figure S1G**). In line with the data on the proliferation at pH 5.5 (**Figures 8A–C**), knockdown of any genes coding the ASIC1a, α-ENaC, and γ-ENaC subunits significantly impaired the anti-migration activity of mambalgin-2 at pH 5.5 (**Figures 8D, E**). Thus, all three ASIC1, α-ENaC, and γ-ENaC subunits are necessary for the mambalgin-2 function in A549 cells at acidic conditions.

### Elevated Expression of ASIC1 in Lung Adenocarcinoma Correlates With Worse Survival Prognosis for Patients

**Figure 9:**
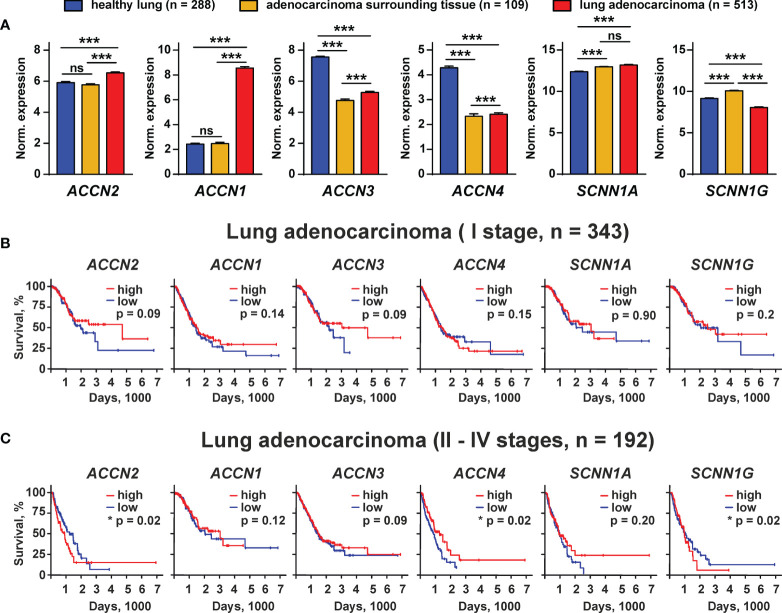
Bioinformatic analysis of the *ACCN2*, *ACCN1*, *ACCN3*, *ACCN4*, *SCNN1A*, and *SCNN1G* expression in healthy people and patients with lung adenocarcinoma from the GTEX and TCGA LUAD databases. **(A)** Analysis of the gene expression in healthy lung biopsies (GTEX study) and in samples of lung adenocarcinoma surrounding tissue, and lung adenocarcinoma specimen (TCGA LUAD study). Data are presented as normalized gene expression ± SEM; *** (p < 0.001) indicates significant difference between the data groups by one-way ANOVA followed by Tukey’s *post-hoc* test. **(B, C)** Kaplan–Meier analysis of the correlation between survival of the patients with lung adenocarcinoma at I **(B)** and II–IV stages **(C)** and different expressions of the *ACCN2*, *ACCN1*, *ACCN3*, *ACCN4*, *SCNN1A*, and *SCNN1G* genes. * (p <0.05) indicates significant difference between the overall survival prognosis for patients with the high (above median) and low (below median) gene expressions according to the log-rank test, n.s. - no significant difference (p>0.05).

To evaluate the physiological role of the different members of the DEG/ENaC family in the lung adenocarcinoma progression, we performed a comparative analysis of the expression of the *ACCN2*, *ACCN1, ACCN3, ACCN4, SCNN1A*, and *SCNN1G* genes in human lung adenocarcinoma tissues, tissues surrounding lung adenocarcinoma, and human healthy lung samples from TCGA LUAD and GTEX databases. We found that the *ACCN2* and *ACCN1* expression was elevated in lung adenocarcinoma in comparison to normal lung samples and lung adenocarcinoma surrounding tissue (**Figure 9A**). The expression levels of *ACCN3* and *ACCN4* were significantly lower in adenocarcinoma and surrounding tissue than in healthy lung samples (**Figure 9A**). The expression of mRNA coding the α-ENaC and γ-ENaC subunits was up-regulated in adenocarcinoma-surrounding tissue, but the expression of these mRNAs was up- and down-regulated, respectively, in adenocarcinoma in comparison to normal tissue (**Figure 9A**).

To study the correlation between the expression of the ASIC and ENaC subunits and survival prognosis for the patients with lung adenocarcinoma at different stages of the disease, we performed the Kaplan–Meier analysis of the patient biopsies from the TCGA LUAD database. No correlation was found between the expression of the studied ASIC and ENAC subunits and the survival prognosis for the patients with lung adenocarcinoma at stage I (**Figure 9B**). Analysis of the samples from the patients with adenocarcinoma at stages II–IV by the Kaplan–Meier method showed that the patients with a lower expression of mRNA coding the ASIC1 and γ-ENaC subunits demonstrated the better survival prognosis (**Figure 9C**). In contrast, a lower expression of mRNA coding ASIC4 correlated with the worse survival prognosis (**Figure 9C**). The expression of all other investigated genes did not correlate with the survival prognosis of the patients with lung adenocarcinoma at the stages II–IV. Thus, both ASIC1 and γ-ENaC may be implicated in the lung adenocarcinoma progression. It should be noted that mRNA expression does not necessarily correlate with the protein expression, so we cannot conclude that the ASIC1 and γ-ENaC subunits are really involved in lung adenocarcinoma progression, but the demonstrated phenomenon may point on the existence of a new class of molecular targets for lung cancer therapy.

## Discussion

Acidification of the tumor and its microenvironment drives the growth and metastasis of cancer cells (32). ASICs, the acid-sensitive channels from the DEG/ENaC family, are one of the most abundant sensors of acidification on the plasma membrane and participate in the pathogenesis of many cancers including lung, breast, pancreatic, and hepatocellular carcinomas (3, 18–20). Thus, these channels can be considered prospective targets for cancer therapy. However, a lack of selective and effective inhibitors hampers the targeting of these receptors. Previously, we demonstrated that mambalgin-2 from black mamba *Dendroaspis polylepis* controls the progression of leukemia (15), glioma (16), and melanoma cells (17). Here we studied the effects and molecular targets of mambalgin-2 in lung adenocarcinoma cells and normal lung fibroblasts.

Mambalgin-2 inhibited the growth and migration of A549 and Lewis-P29 cells and growth of WI-38 cells, and more pronounced effects were observed at low pH (**Figures 1A**, **2A, B**, **S2B**, **S3**). No toxin effect in normal HLF fibroblasts is consistent with the previously reported selective action of mambalgin-2 in glioma and melanoma cells, but not in normal astrocytes and keratinocytes (16, 17). This difference in the action points on the existence of a “pharmacological window”—the concentration range—in which mambalgin-2 inhibits the growth of cancer cells without a toxicity for normal ones. This selectivity of the action allowed us to propose the different presentations of the mambalgin-2 targets on the plasma membrane surface of the cancer and normal cells. Indeed, the study of the expression repertoire of the genes coding the ASIC and ENaC subunits revealed that in contrast to lung adenocarcinoma cells, lung normal fibroblasts do not express ASICs and α-ENaCs at all (**Figure 4**). A similar situation is observed in Lewis cells: the parental Lewis lung carcinoma cells resistant to mambalgin-2 do not express ASIC1, while metastatic Lewis cells sensitive to mambalgin-2 express very high amounts of ASIC1. Study of the functionally active acid-sensitive channels in A549, WI-38, and HLF cells confirmed the absence of these channels only in lung normal fibroblasts (**Figures 5B, E, H**). Notably, WI-38 cells, in spite of their common use as a model of normal cells, are transformed by the SV40 virus to increase the possible number of passages, and this transformation may lead to appearance of tumorigenicity by inactivation of the tumor-suppressing p53 and Rb proteins important for the cell cycle control in epithelial cells (33). Thus, this cell line occupies a middle position sharing some properties of cancer and normal cells.

In glioblastoma cells, the ASIC1 subunit can form the heterotrimer with the α- and γ-ENaC subunits (14, 34). Extracellular microenvironment acidification leads to the recruitment of the ASIC1/ENaC channels into the cell membrane (14), and the inward cation current mediated by these heteromeric channels may drive the glioma cell growth and migration (35). Previously, we demonstrated that mambalgin-2 extracts the ASIC1a and α-/γ-ENaC subunits from the membrane fraction of the patient-derived melanoma cells (17). Here, we showed the mambalgin-2 binding to the ASIC1a, α-ENaC, and γ-ENaC subunits extracted from the membrane fraction of A549 cells (**Figures 6A–C**) and the formation of the ASIC1a/α-ENaC/γ-ENaC complex (**Figures 6D, E**). Analysis using siRNA confirmed that the all three subunits are important for mambalgin-2’s effect on the viability and migration of A549 cells at pH 5.5 (**Figure 8**).

For the first time, we studied the mambalgin-2 influence on the acidification-evoked currents through the ASIC1a/α-ENaC/γ-ENaC heterotrimeric channels (**Figure 7**). It was revealed that the toxin inhibits the heteromeric channels much more effectively than ASIC1a homomeric ones, while amiloride inhibits both types of the channels with the same efficacy (**Figures 7D, E**). Our results are consistent with the previously published data on the properties of the heterotrimeric ASIC/ENaC complexes (36). It was proposed that the different pharmacological properties of the homomeric and heterotrimeric channels can be explained by the formation of new inter-subunit interactions. No difference in the amiloride inhibitory effect at these channels (**Figure 7E**) also agrees with those data, which suggest that the amiloride binding site within the pore is formed by the single ASIC subunit (36). Interestingly, PcTx1 was shown to be a valuable tool for a functional discrimination between the heteromeric channels with different combinations of the ASIC1a/ASIC2a subunits (37). Here we demonstrated that mambalgin-2, similar to PcTx1, can be used in the same way to discriminate between the homomeric ASIC1a and heteromeric ASIC1a/α-ENaC/γ-ENaC channels.

A stronger interaction with the ASIC1a/α-ENaC/γ-ENaC heterotrimers can explain the more pronounced antiproliferative effect of mambalgin-2 in A549 than in WI-38 cells (**Figures 1A, B**). Indeed, mRNAs coding the ASIC1a, α-ENaС, and γ-ENaC subunits were found in A549 cells, while mRNA for α-ENaС was absent in WI-38 cells. We propose that mambalgin-2 targets the homomeric ASIC1 channels in WI-38 cells, while the toxin effect in A549 cells can be related both with the homomeric ASIC1 and heteromeric ASIC1a/α-ENaC/γ-ENaC channels. In line with it, inhibition of ASIC1 by PcTx or the *ACCN2* gene knock-down both significantly reduced the migration of A549 cells (3).

Previously, we showed that mambalgin-2 causes the cell cycle arrest in the G1 and S phases for leukemia and glioma cells (15, 16), but in A549 and Lewis-P29 cells it causes the cell cycle arrest in the G2/M phase (**Figures 3A, B**). This points on the possible implication of different intracellular mechanisms for control of the growth of leukemia/glioma and carcinoma cells. Moreover, mambalgin-2 promotes the more prominent cell cycle arrest but less severe apoptosis in Lewis-P29 cells in comparison with A549 cells (**Figures 3** and **S5**), which can also indicate the existence of different intracellular pathways and/or molecular targets in cancer cells modulated by mambalgin-2. Additionally, mambalgin-2 inhibited the proliferation of A549 cells even at pH 7.4 (**Figure 1A**) and the knockdown only of the ASIC1 expression abolished the toxin effect at normal pH (**Figure 8A**). Possibly, mambalgin-2 can bind to the ASIC1a channels in the closed or desensitized states (26, 38) and activate the metabotropic signaling through the channel without the pore opening. In line with this suggestion, it was shown that the intracellular domain of the ASIC channels can interact with different intracellular signaling messengers (39). 

Bioinformatic analysis of the gene expression in the tissues from the patients with lung adenocarcinoma revealed possible implications of ASIC1, ASIC2, ASIC3, ASIC4, α-ENaC, and γ-ENaC in the disease progression (**Figure 9A**). Indeed, all these channels are involved in acidosis-induced tumor progression: ASIC1 and α-ENaC mediate the epithelial–mesenchymal transition of pancreatic cancer (19, 40), while ASIC2 promotes the invasion and metastasis of colorectal cancer (41). Interestingly, ASIC3, which is down-regulated in lung adenocarcinoma samples, may also drive the migration of pancreatic cancer cells (19), so its role in cancer progression may depend on the carcinoma type. Notably, ASIC4 may down-regulate the ASIC1a expression on the cell surface (42). Thus, its down-regulation in lung carcinoma samples may be linked with the up-regulation of ASIC1. In line, the low expression of the gene coding ASIC1 and the high expression of the gene coding ASIC4 are correlated with better survival prognosis for the patients with lung adenocarcinoma at II–IV stages (**Figure 9C**). Overexpression of γ-ENaC was previously described for cervical cancers (43). Observed here, the lower expression of γ-ENaC in lung adenocarcinoma in comparison with normal lungs (**Figure 9A**) and the correlation of the increased γ-ENaC expression with worse survival prognosis (**Figure 9C**) may reflect the need to maintain the proper ratio between the ASIC homomers and ASIC1/α-ENaC/γ-ENaC heteromers in tumors and requires an additional detailed study. Notably, the bioinformatic analysis only points on the ASIC1- and γ-ENaC-containing channels as on promising targets for lung cancer, but further study is required to confirmthe actual implication of these channels in lung carcinoma progression.

The behavior of cells in a monolayer is significantly different from that of cells in bulky tumors. This is due to intercellular interactions, differences in pH, and different expression levels of the molecules responsible for the cell proliferation inside and outside the tumor (44). Moreover, signaling pathways can differ in bulky tumors and cell monolayers, which leads to the development of resistance to chemotherapeutic substances (45). Here, we showed that mambalgin-2 inhibits the growth of the multicellular spheroids reconstructed from A549 cells (**Figures 1D, E**). Thus, it can bypass the resistance mechanisms of the bulky tumors at least in *in vitro* experiments. Interestingly, previously we showed that another three-finger protein SLURP-1 inhibits the growth of the spheroids reconstructed from A549 cells (46). Probably, a three-finger fold can be used for the design of novel channel-targeting anticancer drugs.

In summary, the selective inhibition of the growth and migration of lung adenocarcinoma cells by mambalgin-2 was shown. For the first time, interaction of mambalgin-2 with the ASIC1a/α-ENaC/γ-ENaC heterotrimeric channels was confirmed by both the affinity chromatography in lung adenocarcinoma cells and electrophysiology experiments in *X. laevis* oocytes. Relations between the ASIC1a, α-ENaC, and γ-ENaC expression and mambalgin-2 effects in cancer cells were revealed. The expression of the heteromeric ASIC1a/α-ENaC/γ-ENaC channels can be considered a marker of the cell oncogenicity.

## Data Availability Statement

The original contributions presented in the study are included in the article/**Supplementary Material**. Further inquiries can be directed to the corresponding author.

## Author Contributions

Conceptualization: AS, MB, EL; data curation: AS, VC-N, MB, DK, EL; formal analysis: AS, VC-N, MB, DK, EL; funding acquisition: EL, MK; investigation: AS, VC-N, MB, DK, MS, OS; methodology: AS, VC-N, MB, DK, SK, MS, OS, EL; project administration: EL, MK; resources: AS, EL, MK; software: AS, VC-N, MB, DK, SK, MS; supervision: EL, visualization: AS, VC-N, MB, EL; writing—original draft preparation: AS, MB, EL; writing—review and editing: VC-N, DK, SK, MS, MK. All authors contributed to the article and approved the submitted version.

## Funding

This research was funded by the grant from the Ministry of Science and Higher Education of Russian Federation (project № 075-15-2020-773).

## Conflict of Interest

The authors declare that the research was conducted in the absence of any commercial or financial relationships that could be construed as a potential conflict of interest.

## Publisher’s Note

All claims expressed in this article are solely those of the authors and do not necessarily represent those of their affiliated organizations, or those of the publisher, the editors and the reviewers. Any product that may be evaluated in this article, or claim that may be made by its manufacturer, is not guaranteed or endorsed by the publisher.
