# Supplementary material for: Mambalgin-2 Inhibits Lung Adenocarcinoma Growth and Migration by Selective Interaction With ASIC1/α-ENaC/γ-ENaC Heterotrimer
Source: Front Oncol. 2022 Jun 28;12:904742. doi: 10.3389/fonc.2022.904742 (PMC9273970; doi:10.3389/fonc.2022.904742)
Supplement: Supplementary file 1 [file DataSheet_1.docx]

Supplementary Material

Mambalgin-2 inhibits lung adenocarcinoma growth and migration by selective interaction with ASIC1/α-ENaC/γ-ENaC heterotrimer

Anastasia V. Sudarikova^1,2,†^, Maxim L. Bychkov^1,†^, Dmitrii S. Kulbatskii^1,†^, Vladislav I. Chubinskiy-Nadezhdin^1,2^, Olga V. Shlepova^1,3^, Mikhail A. Shulepko^1^, Sergey G. Koshelev^4^, Mikhail P. Kirpichnikov^1,5^, Ekaterina N. Lyukmanova^1,3,5,*^.

^1^Laboratory of bioengineering of neuromodulators and neuroreceptors, Shemyakin-Ovchinnikov Institute of Bioorganic Chemistry, Russian Academy of Sciences, 117997, Moscow, Russia.

^2^ Group of Ionic Mechanisms of Cell Signaling, Department of Intracellular Signaling and Transport, Institute of Cytology, Russian Academy of Sciences, 194064, St-Petersburg, Russia.

^3^Phystech School of Biological and Medical Physics, Moscow Institute of Physics and Technology (National Research University), 141701, Dolgoprudny, Moscow Region, Russia

^4^Laboratory of neuroreceptors and neuroregulators, Shemyakin-Ovchinnikov Institute of Bioorganic Chemistry, Russian Academy of Sciences, 117997, Moscow, Russia.

^5^Interdisciplinary Scientific and Educational School of Moscow University «Molecular Technologies of the Living Systems and Synthetic Biology», Faculty of Biology, Lomonosov Moscow State University, 119234, Moscow, Russia.

*** Correspondence:**Corresponding Author
ENL: [ekaterina-lyukmanova@yandex.ru](mailto:ekaterina-lyukmanova@yandex.ru)

^†^ These authors have contributed equally to this work and share first authorship

# Supplementary Tables

**Supplementary Table 1. The siRNA sequences for knock-down of the *ACCN2*, *SCNN1A*, and *SCNN1G* genes**

| **Gene** | **siRNA sequence** | |
| --- | --- | --- |
|  | **sense** | **antisense** |
| *ACCN2* | AUAUAGAAAUAGAAAAUGCAG | GCAUUUUCUAUUUCUAUAUGA |
|  | UUUUGUUAAAUGUAAAAAGAA | CUUUUUACAUUUAACAAAACU |
|  | UAUGUAUUGCUCAGAUUUGUU | CAAAUCUGAGCAAUACAUAGG |
| *SCNN1A* | ACAUAAACGGGCAAGAUUCAG | GAAUCUUGCCCGUUUAUGUAU |
|  | UGUUCUUGGAGCAACUUCCUG | GGAAGUUGCUCCAAGAACAGU |
|  | UGUUGUUGCAGAAGAACUCGA | GAGUUCUUCUGCAACAACACC |
| *SCNN1G* | AAAUUGAAUAACUAAAUCCAU | GGAUUUAGUUAUUCAAUUUUG |
|  | AGAUUCUUCUUGAUUUUGGCU | CCAAAAUCAAGAAGAAUCUGC |
|  | UCUUUUGCUAUUUAAAAGCAA | GCUUUUAAAUAGCAAAAGAAG |
| Scramble | UUCUCCGAACGUGUCACGU | ACGUGACACGUUCGGAGAA |
|  | GAAAUUUAUACAAACUAUCAA | UUGAUAGUUUGUAUAAAUUUC |
|  | GGAGAUAAUAACGAAAUAUAA | UUAUAUUUCGUUAUUAUCUCC |

# Supplementary Table 2. Primers used for real-time PCR

| **Gene** | **Primer** | | **Amplicon size, bp** |
| --- | --- | --- | --- |
|  | **Forward** | **Reverse** |  |
| β*-actin* | CATGTACGTTGCTATCCAGGC | CTCCTTAATGTCACGCACGAT | 88 |
| *GPDH* | ACAACTTTGGTATCGTGGAAGG | GCCATCACGCCACAGTTTC | 73 |
| *RPL13a* | TCAAAGCCTTCGCTAGTCTCC | GGCTCTTTTTGCCCGTATGC | 104 |
| *ACCN2 (ASIC1)* | CGAAGCAGGCATCAAAGTGC | TTTGGATGATAGGGAGCCACG | 642 |
| *ACCN1 (ASIC2)* | CACCAAGACTTCACCACAGTGTTT | TGTAGCGGGTCTCACAGTCA | 409 |
| *ACCN3 (ASIC3)* | TACAGAACTGTGCCCACCC | GGTCTTCGGAACAGAGCAGA | 502 |
| *ACCN4*  *(ASIC4)* | GAGGAGAGAGACAAGCGGCA | GTCCAGCATGATCTCCAGGC | 930 |
| *SCNN1A*  *(α-ENaC)* | CCAGGCCGCTGCACCT | GCCGATCTTCCAGTCCTTCC | 750 |
| *SCNN1G*  *(γ-ENaC)* | GAGTGACGTGCCAATCAGGA | TCTCCGAAACCACAGATGGC | 305 |

# Supplementary Figures


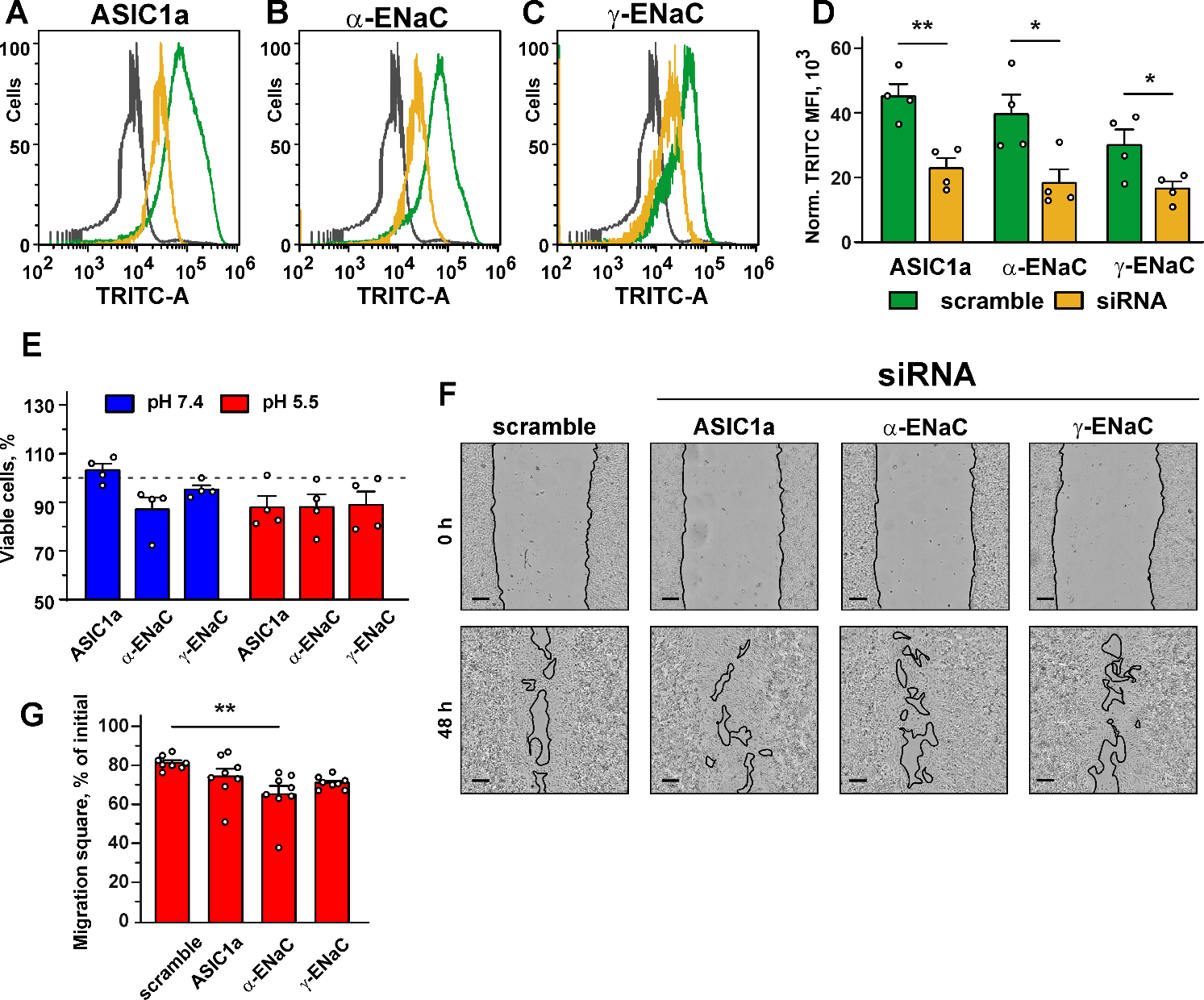


**FIGURE S1. Influence of knock-down of the ASIC1a, α-ENaC, and γ-ENaC genes on proliferation and migration of A549 cells**. The representative cell distribution histograms after transfection of the cells by scramble siRNA and ASIC1a **(A)**, α-ENaC **(B)**, and γ-ENaC **(C)**-specific siRNA and quantification of the ASIC1a, α-ENaC, and γ-ENaC expression after siRNA transfection **(D)**. Data presented as normalized MFI ± SEM (n = 4). * (p < 0.05) and ** (p < 0.01) indicate significant difference between the data groups by the two-tailed t-test; **(E).** Influence of the ASIC1a, α-ENaC, and γ-ENaC knock-down on viability of the A549 cells. Data are % of the cells incubated with scramble siRNA (dashed line) ± SEM (n = 4). **(F).** Representative pictures of wounds for A549 cells incubated with scramble or ASIC1a, α-ENaC, and γ-ENaC-specific siRNAs at pH 5.5. Cells were incubated 48 h. Scale bar = 100 µm. **(G).** Wound area occupied by migrating A549 cells after ASIC1a, α-ENaC, and γ-ENaC knock-down. Data are presented as % of the wound surface, occupied by migrating cells ± SEM (n = 8), ** (p<0.01) indicates significant difference between the data groups by one-way ANOVA followed by Dunnet’s *post hoc* test.


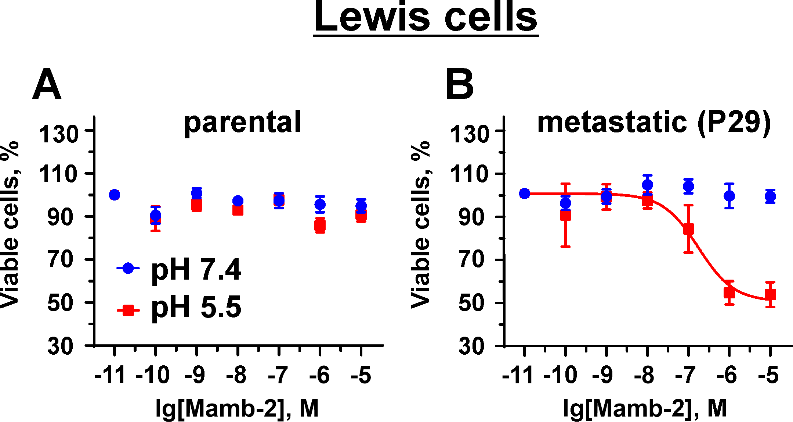


**FIGURE S2. Dose-response effects of mambalgin-2 on viability of Lewis lung adenocarcinoma cells (A) and its metastatic P29 subline (B) cultured at pH 7.4 and 5.5**. Data are presented as % of the control (untreated cells) ± SEM (n = 4).


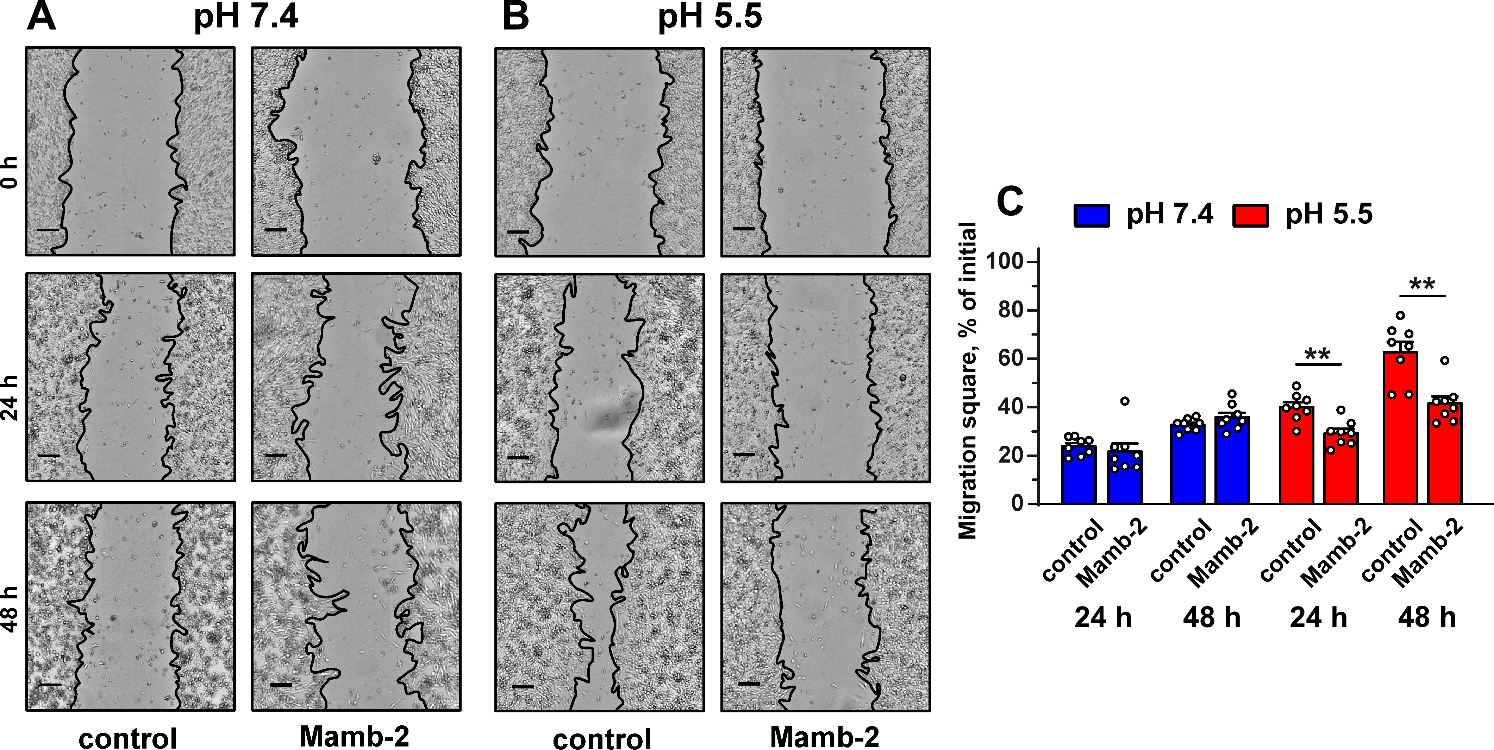


**FIGURE S3. Influence of mambalgin-2 on migration of** **Lewis-P29 cells cultivated at pH 7.4 and 5.5** **(A,B).** Representative pictures of wounds for metastatic Lewis cells incubated at pH 7.4 **(A)** and at pH 5.5 **(B)** in absence or presence of 1 µM of mambalgin-2. Cells were incubated with mambalgin-2 for 24 h and 48 h. Scale bar = 100 µm. **(C)**. Wound area occupied by migrating Lewis-P29 cells. Data are presented as % of the wound surface, occupied by migrating cells ± SEM (n = 8), ** (p<0.01) indicates significant difference between the data groups by two-tailed t test.


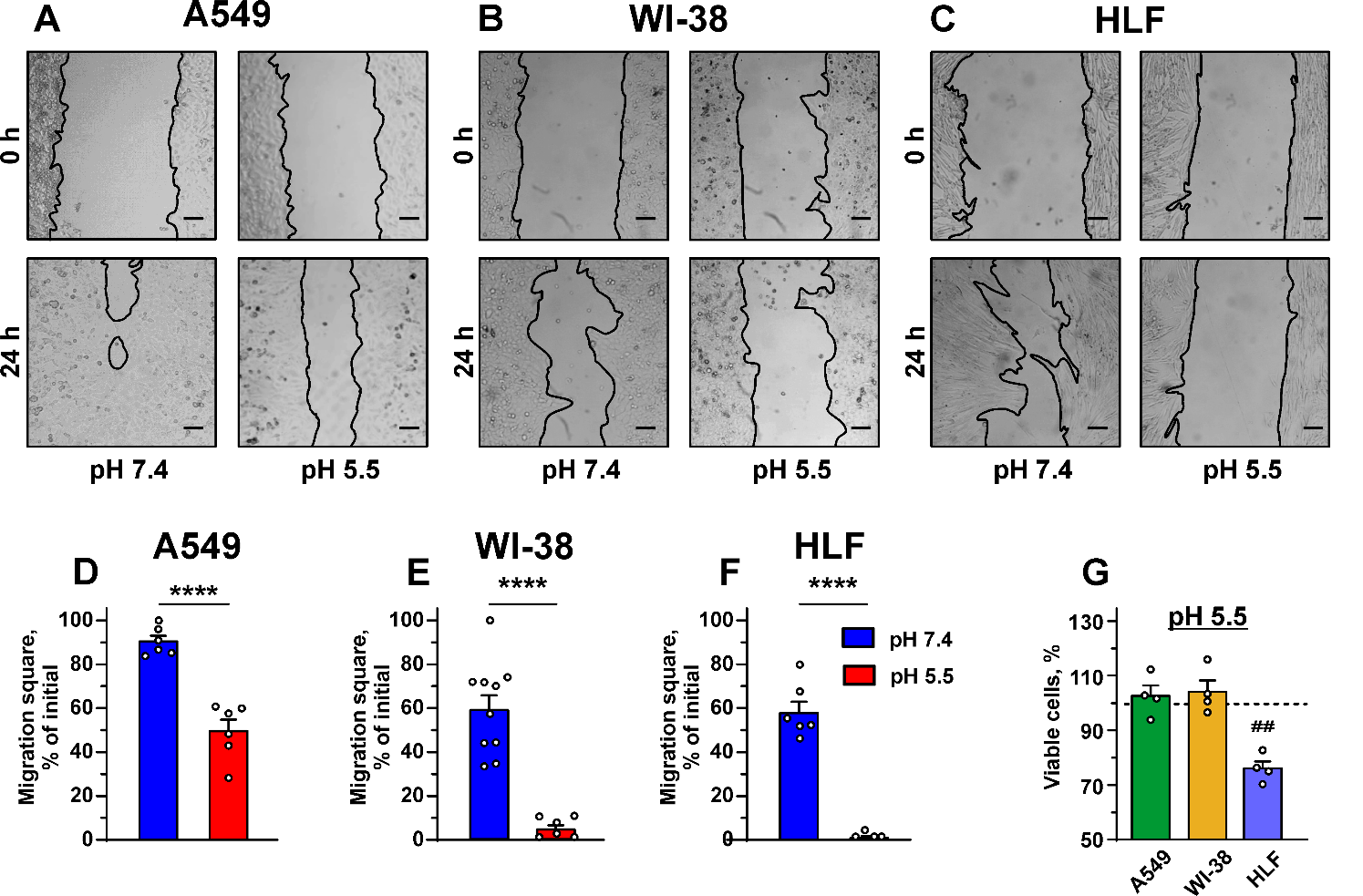


**FIGURE S4. Influence of media acidification from pH 7.4 to pH 5.5 on the motility and viability of A549, WI-38, and HLF cells.** (A-C**)** Representative pictures of wounds for A549 **(A)**, WI-38 **(B)**, and HLF **(C)** cells. Scale bar = 100 µm. **(D-F)** Wound area occupied by migrating A549 **(D)**, WI-38 **(E)**, and HLF **(F)** cells. Data are presented as % of the wound surface occupied by migrating cells ± SEM (n = 6), *** (p<0.001) indicates significant difference between the data groups by Welch’s two-tailed t-test. **(G)** Effect of media acidification on the viability of lung cells. Data presented as % of viable cells cultivated at pH 5.5 from the control cells cultivated at pH 7.4 ± SEM (n = 4). ## (p < 0.01) indicates significant difference from control cells according to one-sample t-test.


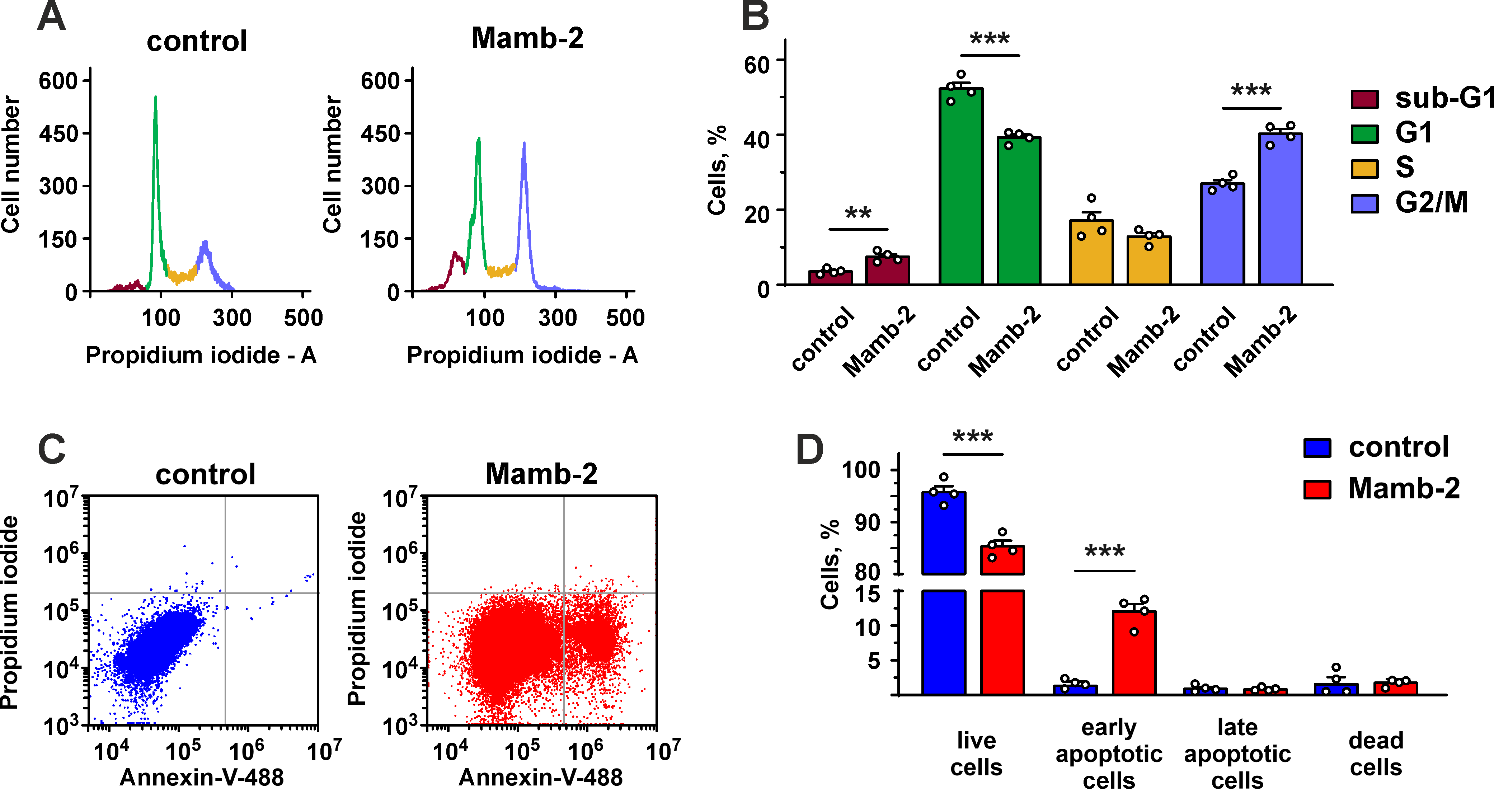


**FIGURE S5. Analysis of cell cycle and apoptosis induction by mambalgin-2 in Lewis-P29 cells**. **(A)** Representative nuclei population distribution of Lewis-P29 cells incubated in absence (control) or presence of 1 µM mambalgin-2. **(B)** % of cells in each cell cycle phase. Data are presented as % of cells in each cell cycle phase ± SEM (n = 4). ** (p < 0.01) and *** (p < 0.001) indicate significant difference between the data groups by two-tailed t-test. **(C)** Representative pictures of phosphatidylserine externalization analysis upon the 1 µM mambalgin-2 treatment of Lewis-P29 cells by flow cytometry with Annexin V-488 and Propidium iodide (control is without mambalgin-2). **(D)** Percentage of Lewis-P29 cells with externalized phosphatidylserine and bound propidium iodide in absence (control) or presence of 1 µM mambalgin-2. The data are presented as % of live, early apoptotic, late apoptotic, and dead cells ± SEM (n = 4). *** (p < 0.001) indicates the significant difference between the data groups by a two-tailed t-test.


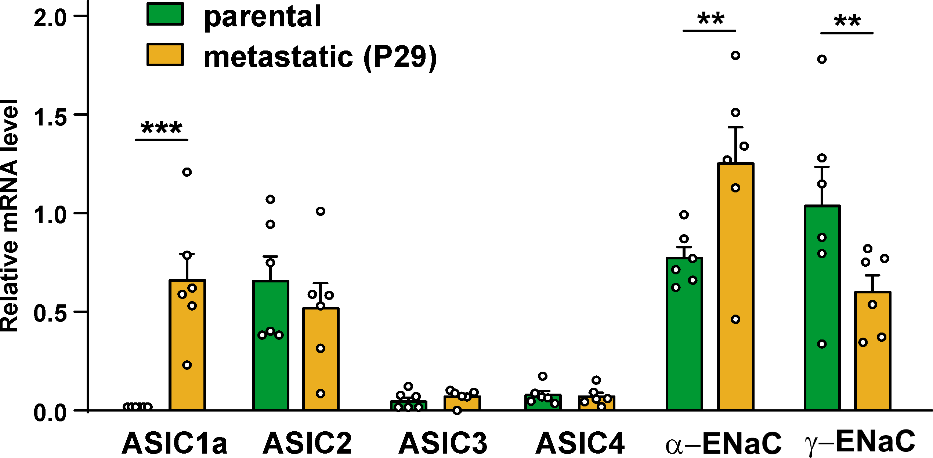


**FIGURE S6. qPCR analysis of the *ACCN2, ACCN1, ACCN3, ACCN4, SCNN1A* and *SCNN1G* expression in Lewis and Lewis-P29 cells**. Gene expression was normalized to the *β-ACTIN, GPDH,* and *RPL13a* housekeeping genes and presented as relative mRNA level ± SEM (n = 6). ** (p < 0.01) and *** (p < 0.001) indicate significant difference between the data groups by a two-tailed t-test.


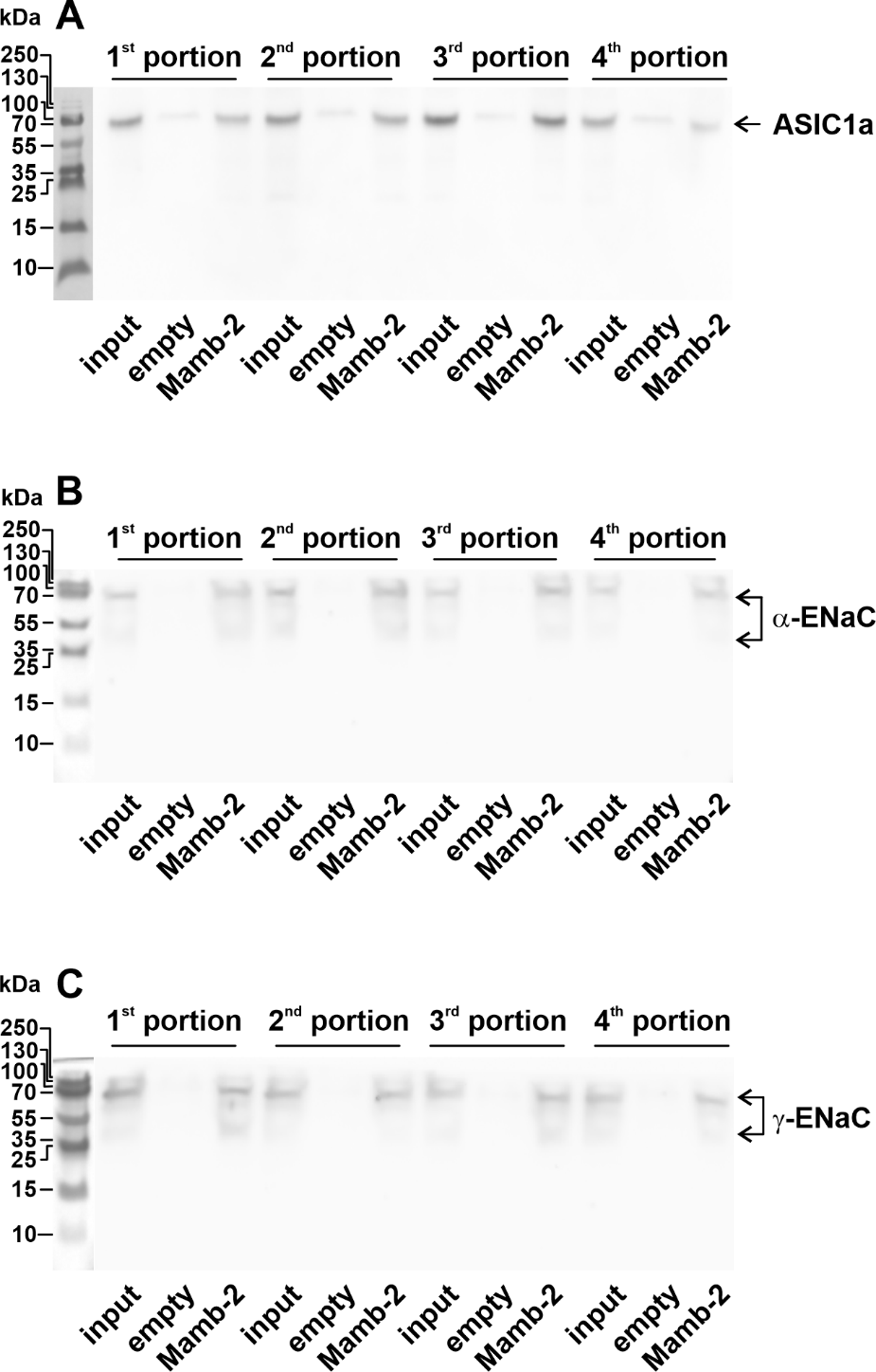


**FIGURE S7.** **Whole membranes used for the analysis of the molecular targets extracted by mambalgin-2 from membrane fraction of A549 cells**. The membranes stained by specific antibodies to ASIC1a (A), α-ENaC (B), and γ-ENaC (C) are shown.


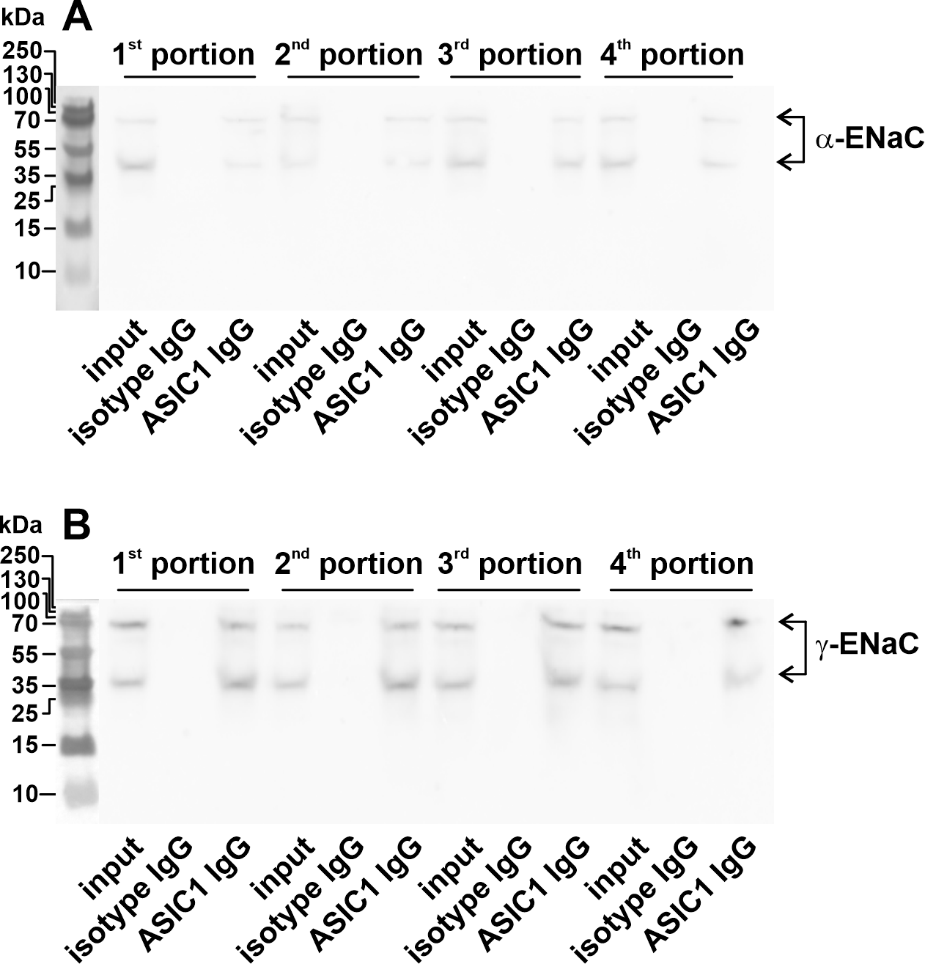


**FIGURE S8.** **Whole membranes used for the analysis of ENaC subunits co-extracted with ASIC1 during immunoprecipitation from membrane fraction of A549 cells by Protein A- agarose conjugated with anti-ASIC1a antibody.** The membranes stained by specific antibodies to α-ENaC (A) and γ-ENaC (B) are shown.


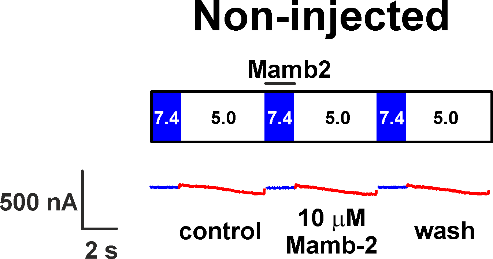


**FIGURE S9.** **Analysis of mambalgin-2 action on non-injected** *Xenopus laevis* oocytes in the two-electrode configuration of the patch clamp technique. The pre-incubation with mambalgin-2 was 15 s (shown off time scale), the stimulation phase (pH 5.0) was 7 s, the recovery phase is not shown.
